# Warburg-like metabolic transformation underlies neuronal degeneration in sporadic Alzheimer’s disease

**DOI:** 10.1016/j.cmet.2022.07.014

**Published:** 2022-09-06

**Authors:** Larissa Traxler, Joseph R. Herdy, Davide Stefanoni, Sophie Eichhorner, Silvia Pelucchi, Attila Szücs, Alice Santagostino, Yongsung Kim, Ravi K. Agarwal, Johannes C.M. Schlachetzki, Christopher K. Glass, Jessica Lagerwall, Douglas Galasko, Fred H. Gage, Angelo D’Alessandro, Jerome Mertens

**Affiliations:** 1 Neural Aging Laboratory, Institute of Molecular Biology, CMBI, Leopold-Franzens-University, Innsbruck 6020, Austria; 2 Laboratory of Genetics, The Salk Institute for Biological Studies, La Jolla, CA 92037, USA; 3 Department of Biochemistry and Molecular Genetics, University of Colorado Anschutz Medical Campus, Aurora, CO 80045, USA; 4 Neuronal Cell Biology Research Group, Eötvös Loránd University, Budapest 1117, Hungary; 5 Department of Molecular and Integrative Physiology, University of Michigan Medical School, Ann Arbor, MI 48109-5624, USA; 6 Department of Cellular and Molecular Medicine, University of California, San Diego, La Jolla, CA 92037, USA; 7 Department of Neurosciences, University of California, San Diego, La Jolla, CA 92037, USA

**Keywords:** direct conversion, induced neurons, reprogramming, Warburg effect, cancer, Alzheimer's disease, pyruvate kinase M, WGCNA, metabolomics

## Abstract

The drivers of sporadic Alzheimer’s disease (AD) remain incompletely understood. Utilizing directly converted induced neurons (iNs) from AD-patient-derived fibroblasts, we identified a metabolic switch to aerobic glycolysis in AD iNs. Pathological isoform switching of the glycolytic enzyme pyruvate kinase M (*PKM*) toward the cancer-associated *PKM2* isoform conferred metabolic and transcriptional changes in AD iNs. These alterations occurred via PKM2’s lack of metabolic activity and via nuclear translocation and association with STAT3 and HIF1α to promote neuronal fate loss and vulnerability. Chemical modulation of PKM2 prevented nuclear translocation, restored a mature neuronal metabolism, reversed AD-specific gene expression changes, and re-activated neuronal resilience against cell death.

## Highlights

• iNs from patients with AD express cancer-associated PKM2
• PKM2 facilitates Warburg-effect-like glycolytic reprogramming of old neurons
• Nuclear PKM2 associates with STAT3 and HIF1α to promote neuronal fate loss in AD iNs
• Modulation of PKM2 with shikonin restores healthy neuronal features

## Graphical abstract

**Figure:**
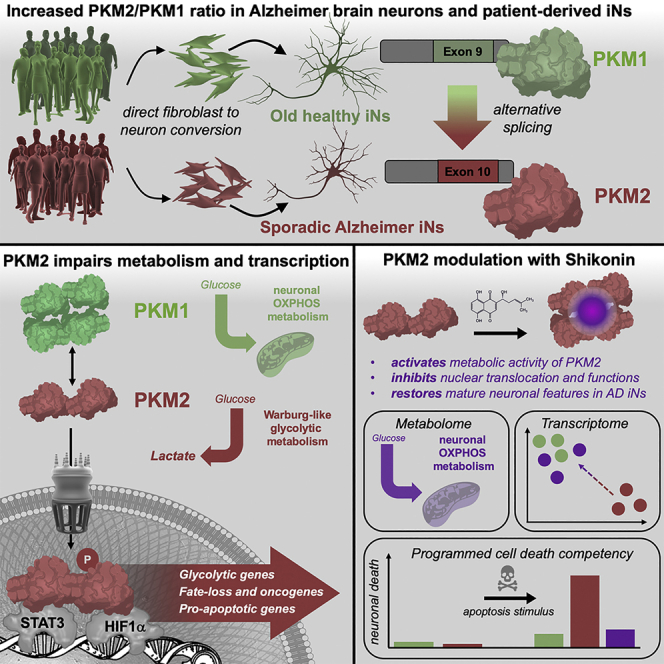

## Teaser

Traxler et al. discover that the metabolic and epigenetic regulator PKM2 is expressed in the brain tissues from patients with Alzheimer’s disease and in patient-derived induced neurons. Neuronal PKM2 causes a metabolic shift, fate loss, and apoptotic competency, which could be partially ameliorated with the PKM2 modulator shikonin, indicating the potential for the therapeutic targeting of PKM2 for age-related neurodegeneration.

## Introduction

Alzheimer’s disease (AD) is the most common form of dementia and a leading cause of death worldwide. The biochemical and cellular changes in AD neurons are still incompletely understood, and this situation is compounded by the paucity of adequate model systems for recapitulating sporadic, age-dependent changes in human cells from patients with AD. Biomarker and postmortem (PM) studies of cerebrospinal fluid (CSF) and human brain tissue have advanced our understanding of sporadic AD pathology; furthermore, transcriptomics, proteomics, and metabolomics are powerful tools to better understand disease-related alterations in human patients (Bai et al., 2020; Higginbotham et al., 2020; Johnson et al., 2020; Seyfried et al., 2017). Besides the typical hallmarks of AD, characterized by aberrant synaptic processes and progressive neuronal death, several proteomic and metabolomic studies have independently identified deficits in splicing and metabolic alterations in AD (Bai et al., 2020; Caldwell et al., 2020; Higginbotham et al., 2020; Johnson et al., 2020; Marinaro et al., 2020; Seyfried et al., 2017). In this regard, elevated levels of metabolic enzymes, such as lactate dehydrogenase A (LDHA) and pyruvate kinase M (PKM), have been suggested to be highly reproducible biomarkers in the CSF of individuals with AD (Higginbotham et al., 2020; Sathe et al., 2019). However, although the identification of potential biomarkers is pivotal, these large-scale human studies are limited in that they only possess a correlative power. To gain a more complete understanding of potential AD-associated transcriptomic and metabolic alterations in neurons, we generated directly converted induced neurons (iNs) from patient-derived fibroblasts by the overexpression of Ascl1:2A:Ngn2 (Mertens et al., 2015). Importantly, iNs maintain the aging signatures of their donors (Huh et al., 2016; Kim et al., 2018; Mertens et al., 2015) and are a unique model system to assess age-related disease phenotypes in live human neurons (Jovičić et al., 2015; Pircs et al., 2021; Victor et al., 2018).

We previously reported that AD-patient-derived iNs lose mature neuronal markers and regress to a hypo-mature state, which in itself parallels the malignant transformation in cancer (Mertens et al., 2021). Understanding the mechanisms that give rise to neuronal hypo-maturity would be invaluable, as it represents an early AD-related phenotype that might be reversible. Neuronal metabolism stands out as a potential convergence platform for aging and disease because multiple disease features of AD, including DNA damage, oxidative stress, dysfunctional enzymes, and cofactors, such as NAD^+^, are all directly linked to the unique metabolic state of postmitotic neurons (Collins et al., 2017; Lautrup et al., 2019; Wang et al., 2021). Here, we identified that PKM2, a key metabolic enzyme and nuclear factor in cancer, is expressed in AD neurons. Through both metabolic and nuclear mechanisms, neuronal PKM2 instates a cellular program that causes a loss of neuronal resilience in AD patient-derived iNs; this program can be ameliorated by chemical PKM2 tetramerization.

## Results

### Functional iNs directly converted from fibroblasts of patients with AD and control donors

**Figure 1:**
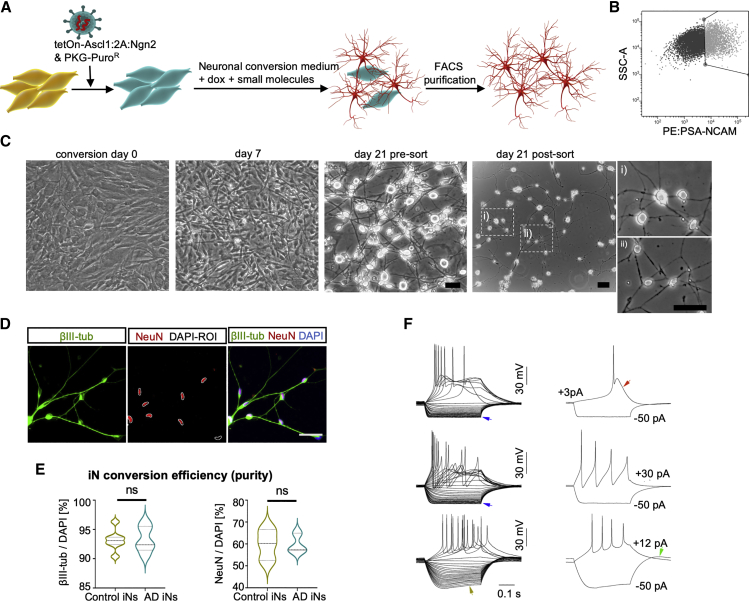
Generation of iNs from patient-derived fibroblasts (A and B) Schematic: generation of iNs from fibroblasts (A) and PSA-NCAM-based FACS purification (B). (C) Phase-contrast images of fibroblasts at day 0 (overconfluent) and at different stages of conversion. Scale bars, 10 μm. (D and E) Immunostainings of βIII-tub and NeuN (D) and quantification of βIII-tub and NeuN-positive cells (E) per DAPI (control, n = 7; AD, n = 5), showing median and quartiles. Significance: unpaired t test, ^∗^p < 0.05 (E). (F) Representative voltage responses of iNs elicited by current step stimulation. Arrows indicate distinctive features associated with specific intrinsic membrane currents. Blue, inward rectification; red, Ca spike; olive, depolarizing voltage sag; green, rebound depolarization.

We obtained punch biopsies and dermal fibroblast cultures from 11 individuals with AD and 11 age-matched, nondemented control donors between 57 and 88 years of age (Figure S1A). The AD group consisted of nine sporadic patients and two patients with familial AD (APP-V717 and PS1-A246E), all of whom received extensive clinical and research characterization and neuropsychological testing (summary data include mini-mental state examination scores [MMSEs]). To minimize potential genetic biases, both control and AD donors were matched according to age, apolipoprotein E (ApoE) genotypes, and sex. Using a transcription-factor-based direct neuronal conversion strategy overexpressing the two pioneer transcription factors Ascl1 and Ngn2, we generated cortical iNs from the donor fibroblasts (Figure 1A). We and others have previously reported that iNs preserve the epigenetic information of their donor ages and reflect an adult-like transcriptomic identity (Huh et al., 2016; Mertens et al., 2015, 2021; Traxler et al., 2019). Following 21 days of conversion, the majority of fibroblasts adopted a mature neuronal morphology, and cells positive for the neuronal surface marker PSA-NCAM were isolated by fluorescence-activated cell sorting (FACS) and re-plated on Geltrex-coated culture substrates (Figures 1B and 1C). Consistently, 93.2% ± 1.8% of the cells in purified iN cultures were positive for βIII-tub, and 59.8% ± 7.0% were neuronal nuclei (NeuN) positive (Figures 1D and 1E). Notably, iNs from all control and AD donors could be enriched to equally high purities and spontaneously developed synapse-like structures marked by the co-expression of synapsin and postsynaptic density protein 95 (PSD95) (Figure S1B). Electrophysiological analysis of iNs from both groups revealed mature physiological properties and strong intrinsic excitability, and many iNs displayed voltage responses with characteristic features that indicated the action of specific voltage-activated membrane currents. Such features included inward rectification mediated by voltage-activated K_ir_ currents, depolarizing voltage sag mediated by hyperpolarization-activated cation currents (I_h_), calcium spikes, and rebound depolarization mediated by low-threshold Ca currents (Figure 1F).

### Gene co-expression modules overlapping between AD iNs and postmortem AD brains point toward aberrant metabolic regulation

**Figure 2:**
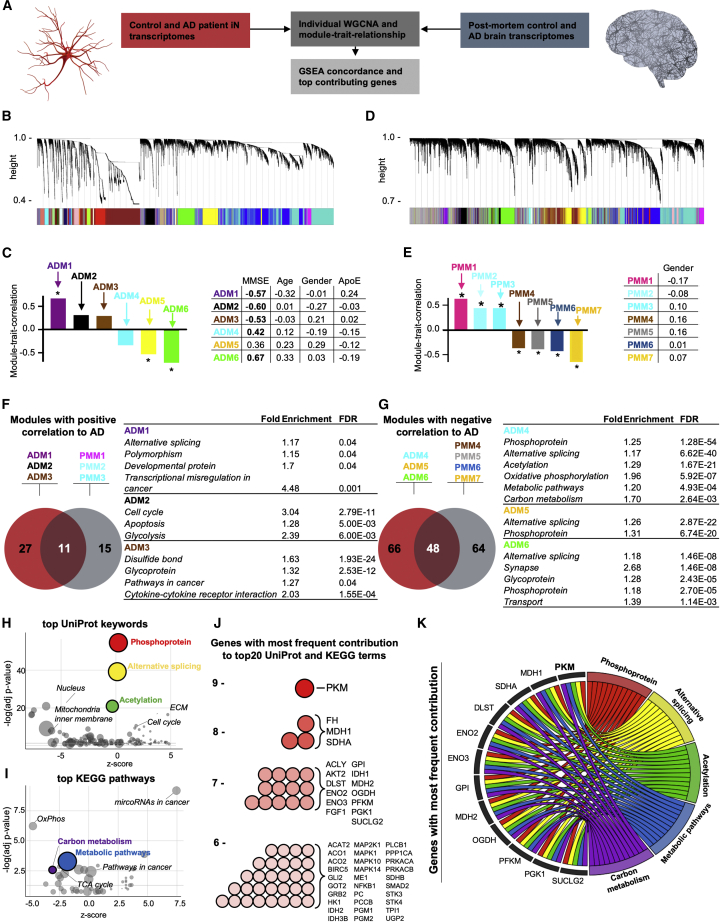
Gene expression network analysis of AD iNs and postmortem brain (A) WGCNA analysis of transcriptomes of control and AD iNs (E-MTAB-10344) and postmortem (PM) brain transcriptomes (GSE5281). (B and D) Cluster dendrograms representing groups of genes identified using WGCNA in iN (B) and PM (D) datasets with the assigned module colors. (C and E) Module-trait relationship of the significant modules correlating to AD and MMSE in iNs (ADMs, AD modules) (C) and modules correlating to AD in PM tissue (PMMs, postmortem modules) (E), with correlation values to MMSE, age, gender, and ApoE genotype. Asterisks and bold values represent significant (p < 0.05) values. (F and G) Enriched GO terms in modules significantly correlated positively (F) or negatively (G) to AD in cultured iNs and PM brain tissue, with the top common GO terms presented in the table. KEGG pathways are displayed in italic. (H and I) UniProt keywords (H) and KEGG pathways (I) according to their adjusted p value (y axis) and nonstatistical *Z* score calculated by GoPlot (x axis). The area of each circle represents the number of genes of that pathway. (J) Most abundant genes of top 10 UniProt keywords and top 10 KEGG pathways. (K) Chord plot showing the 17 genes that are present at least seven times in the 20 pathways and pathways that contain at least 14 of these genes.

To explore the AD-related changes in the gene expression patterns in patient-derived iNs, we performed weighted gene correlation network analysis (WGCNA) on the whole-transcriptome RNA-seq data of FACS-purified iNs from all control and AD subjects (n = 21) (Figure 2A). The resulting co-expression network revealed 29 distinct gene modules representing genes with similar expression changes across all samples (Figure 2B). We were particularly interested in those modules that showed clear, interpretable enrichment for biological functions, so we performed a gene set enrichment analysis (GSEA) that preserved 14 modules holding significantly enriched gene ontology profiles. Module-trait-relationship analysis allowed us to identify six modules that showed a highly significant correlation with diagnosis (AD or control) and the subjects’ cognitive abilities (MMSE); we refer to these as the AD iN modules ADM1–ADM6 (Figures 2C, S1C, and S1D). Notably, ADM1–3 showed a positive correlation with AD diagnosis, whereas ADM4–6 showed a negative correlation. None of the modules appeared to be skewed by donor age, gender, or ApoE genotype, as no significant correlation was detected (Figures 2C and S1E–S1G).

We next sought to investigate the extent to which AD-related gene module expression in iNs reflected the human *in vivo* condition by calculating reference modules based on transcriptomic data from 30 PM hippocampal tissue samples from patients with AD and controls (Figure 2D). Module-trait-relationship analyses resulted in 20 distinct co-expression modules, seven of which were identified as AD PM modules (PMM1–PMM7). Of these, PMM1–3 showed significant positive correlation and PMM4–7 showed a significant negative correlation with AD diagnosis (Figures 2E and S1H). Similar to the iN model, none of the seven PMMs showed a significant correlation with gender as a potentially confounding factor (Figures 2E and S1I). Importantly, parallel GSEA of ADM1–6 and PMM1–7 suggested a substantial functional overlap between the iN and PM brain AD modules. Of the pathways (KEGG and UniProt keywords) that were significantly enriched within the positively correlating modules ADM1–3, eleven terms (29.0%) were also enriched in PMM1–3, and of the pathways within the negatively correlating modules ADM4–7, 48 terms (42.1%) were also enriched in PMM4–7 (Figures 2F and 2G). As expected, some of the resulting functional gene categories such as cell cycle, pathways in cancer, oxidative phosphorylation, and synaptic transmission corresponded to the hypo-mature neuronal state in AD iNs that we have previously described to be accompanied by less complex neuronal branching morphologies, decreased synaptic densities, and decreased spontaneous network activity (Mertens et al., 2021). Most interestingly, this analysis revealed a transcriptional pattern that points toward aberrant metabolism in AD. The ten most significant UniProt keywords encompassed phosphorylation, alternative splicing, and acetylation (Figure 2H), and the ten most significant KEGG pathways included carbon metabolism and metabolic pathways (Figure 2I). Analysis of the ROSMAP whole-brain PM AD dataset (n = 633) further supported this notion, as the top KEGG pathways and UniProt keywords associated with AD, independent of age and gender, overlapped with our data (Figures S2A–S2C). To identify the individual key regulators that contributed to the AD phenotype in iNs and were detectable in PM brain tissue, we extracted the genes that contributed to these top keywords and pathways with the highest frequency. This analysis revealed that 51 genes contributed to six or more of these terms and that 17 genes contributed to seven or more (Figures 2J and S1J), including enolases (*ENO2* and *ENO3*), glucose-6-phosphate isomerase (*GPI*), malate dehydrogenase (*MDH1*), ATP citrate lyase (*ACLY*), phosphofructokinase muscle (*PFKM*), and phosphoglycerate kinase (*PGK1*). While all of these eight genes encode metabolic enzymes, only the PKM encoded by the *PKM* gene contributed to nine terms. *PKM* also stood out as a major cellular state regulator with nucleocytoplasmic regulation and epigenetic effects through phosphorylation and acetylation (Figures 2K and S1K; Alves-Filho and Pålsson-McDermott, 2016; Yang and Lu, 2013). These data suggest that aberrant metabolic and epigenetic regulation involving *PKM* might drive pathogenic alterations in AD neurons.

### *PKM* isoform switching and a Warburg-like metabolic signature are evident in iNs and postmortem prefrontal cortex tissue from patients with AD

**Figure 3:**
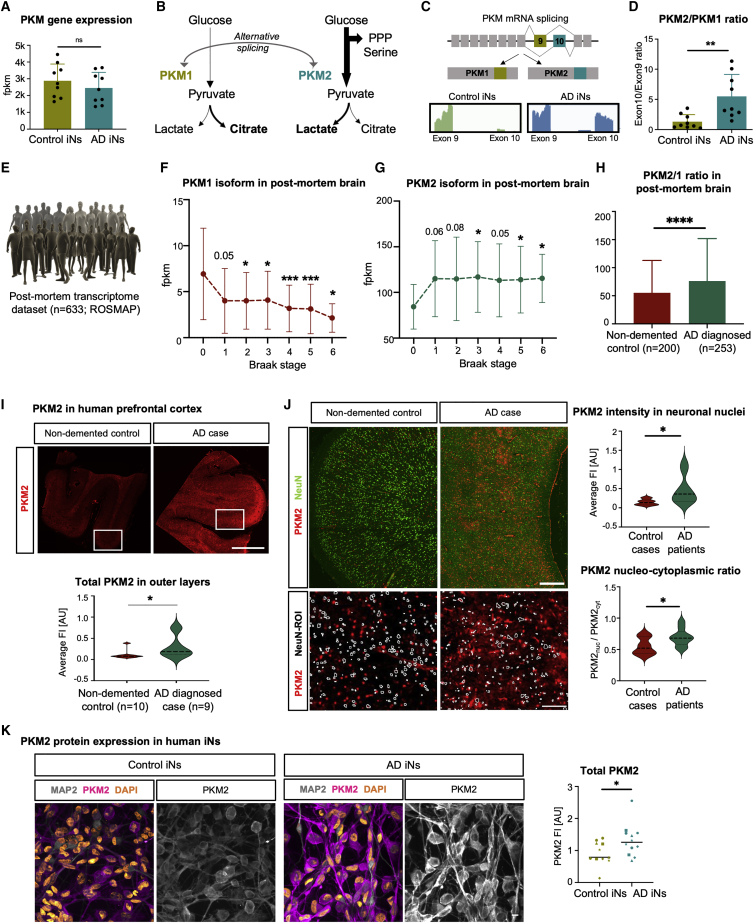
*PKM* isoform switch in AD iNs and postmortem brain tissue (A) RNA-seq counts of *PKM* (control, n = 9; AD, n = 9). (B) Schematic: *PKM* regulation by alternative splicing. (C) RNA-seq reads mapped to exons 9 and 10 of the *PKM*. (D) Bar plot representing exon 10/9 ratios (control, n = 9; AD, n = 9). (E–H) Analysis of ROSMAP bulk transcriptomic dataset (n = 633), divided based on Braak stages (one-way ANOVA, Dunnett’s multiple comparison test, compared with Braak 0, DF 632; PKM1, F = 4.28, p = 0.0003; PKM2, F = 0.84, p = 0.53) or diagnosis after death (Mann-Whitney test). (I and J) Immunostaining of prefrontal cortex of human postmortem brain tissue (control, n = 10; AD, n = 9). Scale bars, 1,000 μm in (I) and 500 and 100 μm in (J). Total PKM2 measured in neuron-rich outer layers (Mann-Whitney test) and in NeuN-ROIs compared with perinuclear regions. (K) Immunostaining and quantification of total PKM2 in MAP2-ROI in control (n = 5) and AD (n = 7) iNs (two independent experiments per donor; shape represents the donor). Scale bars, 10 µm. (A–D and H–N) Bars, mean; error bars, SD; significance, unpaired t test unless otherwise indicated, ^∗^p < 0.05, ^∗∗^p < 0.01, ∗∗∗p < 0.001, ∗∗∗∗p < 0.0001.

PKM has been extensively studied as both a glycolytic enzyme and metabolic master regulator and also as a nuclear factor that contributes to malignant epigenetic transformation in various tumors (Chen et al., 2019; Desai et al., 2014; Gao et al., 2012; Luo et al., 2011; Wang et al., 2014; Yang et al., 2011, 2012a). However, although PKM revealed itself as a central hub gene in our module-trait-relationship analysis in AD iNs, the total mRNA levels of *PKM* showed no differential expression between control and AD iNs (Figure 3A). Notably, PKM activities are critically influenced by alternative splicing, as it contains the two mutually exclusive exons 9 and 10 (Chen et al., 2010). Inclusion of exon 9 forms *PKM1*, the metabolically active isoform that contributes to pyruvate flux to support mitochondrial oxidative phosphorylation (Figure 3B). Alternatively, the inclusion of exon 10 forms the *PKM2* isoform, which has diminished metabolic activity. Imbalance in favor of PKM2 leads to the accumulation of glycolytic metabolites and increased lactate production. This PKM-driven change is a major component of the Warburg effect, which is instrumental in many cancers (Puckett et al., 2021). Interestingly, *PKM* isoform quantification from our paired-end iN RNA-seq data revealed a significant 4.7-fold shift toward *PKM2* splicing in AD iNs, as evidenced by an elevated exon 10 inclusion (Figures 3C and 3D). This change was not present in the donor fibroblasts, suggesting that it is not a carryover from fibroblasts but instead a neuron-specific phenomenon (Figure S2D). Furthermore, increased cancer-like *PKM* isoform switching was also evident in RNA-seq from 633 PM prefrontal cortex samples across Braak stages (De Jager et al., 2018), as *PKM1* levels were decreased and *PKM2* levels increased at the time of death in patients diagnosed with AD (Figures 3E–3G and S2E). Patients with confirmed AD at the time of death ultimately have a significantly increased *PKM2/PKM1* ratio (Figure 3H). Because bulk tissue transcriptome data cannot ensure actual protein changes in neurons, we sought to confirm elevated PKM2 levels in AD PM prefrontal cortex sections (n = 10 healthy control brains and n = 9 brains of patients with sporadic AD). Immunofluorescent analysis revealed elevated levels of total PKM2 immunofluorescence in neuron-rich layers (Figure 3I). The colocalization of NeuN with PKM2 was significantly more likely in AD sections, as PKM2 intensity was 3.1-fold higher in NeuN areas, and interestingly, AD-linked PKM2 was found to predominantly localize to neuronal nuclei over perinuclear regions (Figure 3J). Consistently, AD iNs demonstrated similar 1.5-fold-increased PKM2 protein levels by immunocytochemistry (Figure 3K).

**Figure 4:**
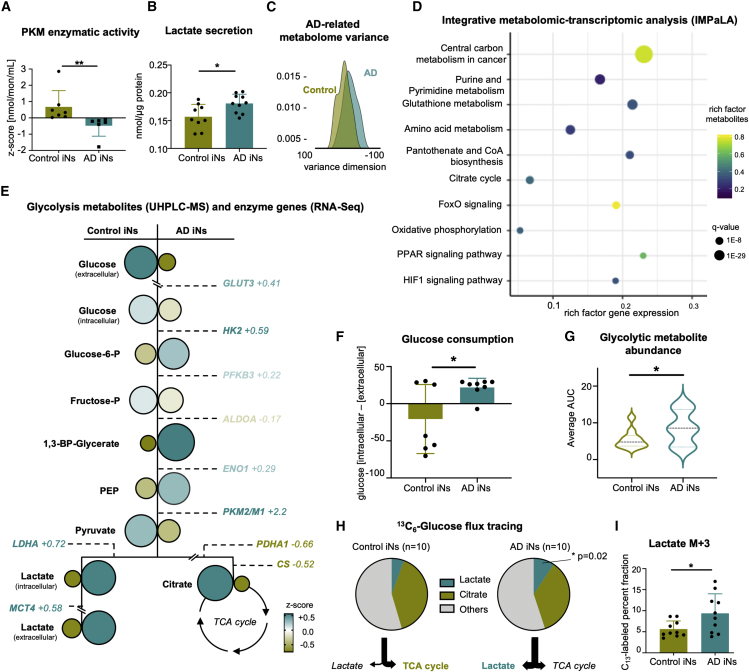
Metabolome reveals Warburg-like metabolic switch in AD iNs (A) Colorimetric assay to determine PKM activity in control (n = 7) and AD (n = 6) iNs (Mann-Whitney test). (B) Colorimetric assay to detect secreted lactate in the supernatant of control (n = 9) and AD (n = 10) iNs. (C) Density plot of PC7 of 160 metabolites measured by UHPLC-MS of control (n = 10) and AD (n = 10) iNs. (D) Integrative transcriptomics and metabolomics (IMPaLa) analysis showing over-represented pathways including gene and metabolites rich factors. (E) UHPLC-MS metabolic landscape in control (left; n = 10) and AD (right; n = 10) iNs. Circles represent *Z* scores of respective metabolites. Size and color of the font indicate the RNA-seq expression levels of related enzymes. (F) Glucose consumption measured as the drop of extracellular glucose after 6 h in culture. (G) Combined averages of all detected glycolytic metabolites in iNs. (H and I) Tracing of isotope-labeled glucose after 6-h incubation with ^13^C_6_-glucose. Fraction of labeled glucose detected in lactate and citrate (n = 10 per group). Dots represent individual donors throughout the figure. Bars, mean; error bars, SD; violin plots, median and quartiles. Significance: unpaired t test, ^∗^p < 0.05, ∗∗p<0.01.

To assess the functional consequences of elevated neuronal *PKM2* mRNA and protein in AD, the metabolic enzymatic activity of PKM in iNs was assessed by measuring the pyruvate levels generated in a certain amount of time using a colorimetric assay. Indeed, AD iNs showed a markedly decreased metabolic PKM activity compared with control iNs (Figure 4A). Further, an accumulation of secreted lactate was consistently evident from colorimetric assays (Figure 4B). To obtain a more detailed understanding of PKM-induced metabolic reprogramming in AD iNs, we performed a semiquantitative ultra-high-performance liquid chromatography-mass spectrometry (UHPLC-MS) metabolomic analysis (De Jager et al., 2018). Evaluation of total UHPLC-MS metabolite levels confirmed a global metabolic switch, as a principal component analysis (PCA) on the 160 metabolites that were reliably detected could clearly separate control and AD iNs along the component PC7, which is functionally enriched for glycolytic pathways and enzymes (Figures 4C, S3A, and S3B). To expose the metabolic pathways most severely impaired by AD-related PKM dysfunction, we performed a multi-omic integration of our RNA-seq transcriptome and UHPLC-MS datasets using the integrative molecular pathway level analysis (IMPaLA) (Kamburov et al., 2011). IMPaLA clearly ranked carbon metabolism as the most critically altered metabolic pathway in AD iNs, as it was scored with the highest significance and highest rich factors for genes and metabolites (Figure 4D). Led by these data, we assessed the individual glycolytic metabolites (UHPLC-MS) and their corresponding canonical glycolytic enzyme genes. Indeed, we detected an increased mRNA abundance of metabolic transporters and enzymes, all of which are consistent with the previously reported LDHA overabundance in AD iNs (Figure 4E; Mertens et al., 2021). Furthermore, the UHPLC-MS metabolome data substantiated a general Warburg-like metabolic switch toward glycolysis in AD iNs as, in addition to lactate secretion, the glycolytic intermediate metabolites glucose-6-phosphate, 1,3-BP-glycerate, phosphoenolpyruvate (PEP), and intracellular lactate levels were increased (Figure 4E). Interestingly, the peak of accumulated glycolytic metabolites was at PEP/1,3-BP-glycerate, thus directly prior to the bottleneck reaction of PKM (Figure 4E). Consistently, we detected a significant increase in glucose uptake in AD iNs (Figures 4F and S4A). Globally, these changes represent a significant increase in all averaged glycolytic metabolites (Figure 4G). Interestingly, and similar to most cancers (Vander Heiden et al., 2009), the glycolytic switch occurs in the absence of global mitochondrial failure, as tracing of ^13^C_6_-glucose demonstrated a stable flux to citrate (Figures 4H, S4B, and S4C). This notion is substantiated by the normal protein levels of the mitochondrial gatekeeper PDH, there being no difference in the total levels of TCA cycle metabolites, the normal mitochondrial membrane potentials, and the unchanged ATP/ADP ratios of the AD neurons (Figures S4D–S4H). Furthermore, oxidative phosphorylation remained intact, as demonstrated by unchanged SDH activity (complex II of ETC; measured as the flux from succinate to fumarate) and there being no differences in mitochondrial respiration between AD and healthy age-matched control iNs (Figures S4I and S4J). Thus, increased lactate production did not occur at the cost of TCA cycle function, as AD iNs appeared to sustain oxidative metabolism and increase their glucose consumption to fuel glycolysis above normal levels. Furthermore, consistent with the mouse models of AD (Hou et al., 2018) and observed increased DNA damage repair in old and AD iNs (Huh et al., 2016; Mertens et al., 2021), AD iNs showed a higher demand for NAD^+^ that was evident from decreased NAD^+^ levels, which likely supported the shift to aerobic glycolysis (Figure S4K). However, while supplementation with nicotinamide riboside (NR) increased the levels of NAD^+^ and precursors, it was not sufficient to reverse glycolytic activity or neuronal lactate secretion (Figures S4L and S4M). Taken together, these data indicate that the isoform switch from PKM1 to PKM2 in AD iNs is associated with a metabolic switch in AD iNs, which shared similarities with the Warburg effect described in many cancers.

### Cancer-like isoform imbalance and nuclear translocation of PKM2 impair the epigenetic landscape of AD iNs

**Figure 5:**
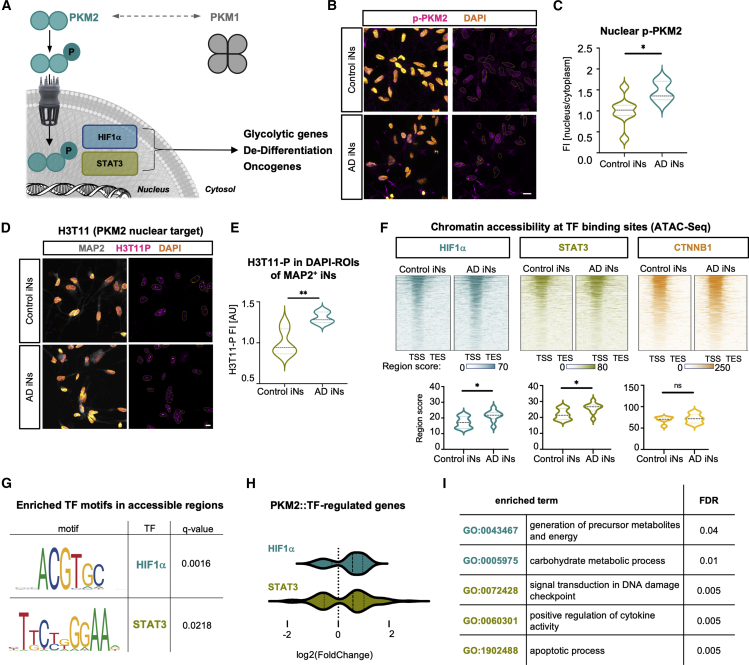
Nuclear PKM2 activity alters the neuronal epigenetic landscape (A) Schematic: phosphorylated PKM2 translocates to the nucleus to interact with transcription factors to regulate gene expression. (B and C) Immunostaining (B) and quantification (C) of p-PKM2(Ser37) (control, n = 8; AD, n = 5). Scale bars, 10 μm. (D and E) Immunostaining (D) and quantification (E) of phosphorylated histone 3 (T11) of MAP2-positive neurons (control, n = 6; AD, n = 5). Scale bars, 10 μm. (F) ATAC-seq profiles around transcriptional start sites of genes regulated by HIF1α, STAT3, and β-catenin (CTNNB1), based on ReMap2020 (control, n = 11; AD, n = 9). (G) HOMER motif enrichment analysis of AD differentially open peaks for HIF1α and STAT3, as previously published (Mertens et al., 2021). (H and I) Differential expression (H) and GO term enrichment (I) of significant genes regulated by HIF1α or STAT3. (B–H) Violin plots: median and quartiles. Significance: unpaired t test, ^∗^p < 0.05, ^∗∗^p < 0.01.

The gene co-expression module analysis of our transcriptome data indicates both a direct metabolic and an indirect nuclear effect of increased PKM2/1 ratios to promote neuronal metabolic reprogramming (Zheng et al., 2016). In addition to a decrease in metabolic PKM activity, alternative splicing of *PKM* in favor of *PKM2* leads to an increased nuclear translocation of PKM. While PKM1 forms a tetrameric complex that cannot enter the nucleus, PKM2 forms dimers that, upon nuclear translocation, acquire protein kinase activity and support the tumor-promoting transcription factors HIF1α, STAT3, and β-catenin (Figure 5A; Alves-Filho and Pålsson-McDermott, 2016; Luo et al., 2011; Yang et al., 2011). The phosphorylation of PKM2 at serine 37 (p-PKM2) is a critical step that triggers its nuclear translocation (Yang et al., 2012b). Immunocytochemical analysis of p-PKM2 revealed pronounced p-PKM2-positive nuclear puncta in the majority of AD iNs, leading to a 1.46-fold increase in p-PKM2 nuclear signal (Figures 5B and 5C). Nuclear p-PKM2 phosphorylates threonine 11 on histone 3 (H3T11-P), and indeed, immunocytochemistry showed bright H3T11-P puncta and a 1.31-fold increased H3T11-P signal in AD iNs, validating an increased protein kinase activity of PKM2 in the nucleus of AD iNs (Figures 5D and 5E). Because collaboration with HIF1α, STAT3, and β-catenin is the key mechanism through which pathogenic nuclear PKM2 promotes cancer transformation, we examined chromatin accessibility around genes regulated by these transcription factors using assay for transposase-accessible chromatin (ATAC) sequencing data from control and AD iNs (n = 20). The integration of iN ATAC data with ChIP-seq data (ReMap2020) for each transcription factor demonstrated increased chromatin accessibility around HIF1α- and STAT3-regulated genes (Figure 5F). No difference in chromatin accessibility was observed at genes regulated by β-catenin. The PKM2 boosting of HIF1α and STAT3 transcriptional activation was further supported by a significant enrichment of HIF1α and STAT3 binding motifs in differentially accessible chromatin regions in AD iNs (Figure 5G; Mertens et al., 2021). Consistently, a substantial majority of 73% of genes regulated by HIF1α and 64% of genes regulated by STAT3 showed an upregulation in mRNA abundance in AD iNs (Figure 5H). GSEA revealed that the genes induced by PKM2::HIF1α were involved in the generation of precursor metabolites and energy and carbohydrate metabolic processes and that the PKM2::STAT3-induced genes promoted damage signaling, cytokine activity, and apoptosis (Figure 5I).

We next isolated neuronal transcriptome changes from the single-nuclei RNA-seq data of PM brain tissue of six healthy control donors and six patients with AD (Figure S6A). These data showed consistently increased signatures for glycolysis, HIF1α signaling, STAT3 signaling, and apoptosis effector genes in the neuronal population (Figure S6B; Grubman et al., 2019). Besides neurons, microglia also switched to glycolysis in AD brains, which is an indicator of microglia activation (Lauro and Limatola, 2020). These data suggest that, in addition to metabolic rewiring, an excess of nuclear PKM2 activities promotes a malignant cellular state in AD iNs and may be how hypo-mature neurons re-instate competency for apoptosis (Kole et al., 2013).

### Induction of aerobic glycolysis in iNs causes immature-like apoptotic competency

**Figure 6:**
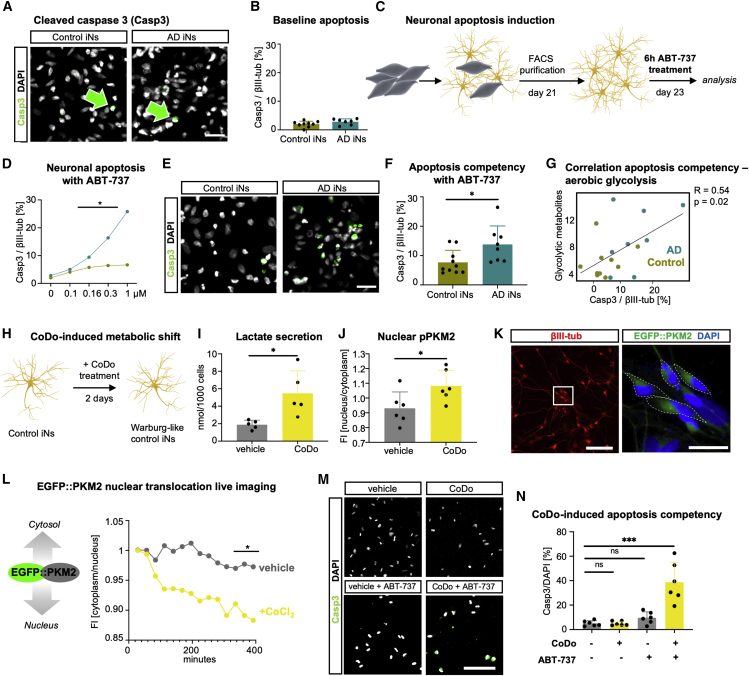
A metabolic shift induces AD-like apoptotic competency in human neurons (A and B) Immunostaining (A) and quantification (B) of cleaved caspase-3 over DAPI of βIII-tub-positive control and AD iNs (control, n = 9; AD, n = 7). Scale bars, 50 μm. Green arrows point out Casp3-positive neurons. (C) Schematic: induction of neuronal apoptosis by ABT-737 treatment. (D) Cell death assessed by cleaved caspase 3/βIII-tub-positive cells of control (green) and AD (teal) iNs in response to 0–1 μM ABT-737. (E and F) Immunostaining (E) and quantification (F) of cleaved caspase-3 in βIII-tub neurons after Bcl2 inhibition in control (n = 10) and AD (n = 8) iNs. Scale bars, 50 μm. (G) Pearson correlation analysis of cleaved caspase-3 immunostaining and glycolytic metabolites (UHPLC-MS). (H and I) Control iNs treated with CoDo for 2 days (H) showed increased lactate secretion (vehicle, n = 5; CoDo, n = 5) (I). (J) Quantification of p-PKM2 FI in the nucleus/cytoplasm comparing vehicle-treated (n = 6) and CoDo-treated control iNs (n = 6). (K) Immunostainings of βIII-tub and EGFP fluorescence of EGFP::PKM2 transduced iNs. Dotted lines show cytoplasmic ROI. Scale bars, 100 and 25 μm. (L) Longitudinal EGFP::PKM2 localization in vehicle-treated (n = 3) or CoCl_2_-treated (n = 4) control iNs. (M and N) Immunostaining (M) and quantification (N) of cleaved caspase-3 positive cells/DAPI before and after ABT-737 (vehicle and CoDo, n = 6; one-way ANOVA, DF: 23, F = 21.34, p < 0.0001, Dunnett’s multiple comparison). Scale bars, 50 μm. Dots represent individual donors throughout the figure. Bars, mean; error bars, SD; significance, unpaired t test, ^∗^p < 0.05, ^∗∗^p < 0.01, ^∗∗∗^p < 0.001.

During later cellular stages of the disease, AD is characterized by extensive neuronal cell death. This is particularly striking because maturing neurons develop efficient apoptotic brakes to avoid neuronal loss over decades of need. Neuronal de-differentiation toward a hypo-mature state might dismantle these apoptotic brakes and contribute to the AD pathophysiology (Arendt et al., 2000; Kole et al., 2013; Mertens et al., 2021). Furthermore, immature neurons respond to glycolytic induction with apoptosis (Bolaños et al., 2010; Herrero-Mendez et al., 2009). Consistently, as evidenced in the longitudinal RNA-seq gene expression profiles from differentiating induced pluripotent stem cell (iPSC)-derived neurons, *PKM2/1* splicing ratios and pro-apoptotic gene expression become strongly suppressed in parallel with neuronal maturation at the same time when anti-apoptotic genes become gradually established (Figure S5; Hollville et al., 2019; Schafer et al., 2019). According to PM brain single-nucleus RNA-seq data, apoptosis is the predominant death pathway in AD neurons, whereas astrocytes and oligodendrocytes show increased gene expression of necroptosis-related genes in AD (Figure S6C). This specificity is clearly mirrored in AD iNs, where the main necroptosis regulators MLKL and RIPK3 are barely detectable at the mRNA level, and no differences were observed between control and AD iNs; however, a more detailed protein analysis is required to evaluate the relative importance of different cell death pathways in AD neurons (Figure S6D). Despite the fact that sporadic AD iNs displayed a hypo-mature state (Mertens et al., 2021) and that the UniProt term apoptosis was significantly enriched in the gene module ADM2, we did not observe considerable fractions of control or AD iNs positive for the apoptotic marker cleaved caspase-3 (Casp3) under standard culture conditions (Figures 6A and 6B). To specifically test for the potential re-gain of apoptotic competency in AD iNs, we exposed iNs to the Bcl2-inhibitor ABT-737, a pro-apoptotic stimulant, and initially monitored a dose-dependent increase in the proportion of cells positive for Casp3 (Figures 6C and 6D). Notably, AD iNs responded with increased cell death compared with control cells to concentrations of 0.16 μM and higher (Figure 6D). We subsequently exposed all control and AD iNs to 0.3 μM ABT-737, which resulted in 3- and 5-fold increases in Casp3 staining in control and AD iNs, respectively (Figures 6E and 6F). Interestingly, the fold increase in Casp3-positive cells significantly correlated with the fold increase of glycolytic intermediates as measured by UHPLC-MS, suggesting that the metabolic switch in neurons is directly tied to their hypo-mature apoptotic competency (Figure 6G).

To determine whether a metabolic shift in AD neurons was directly responsible for their apoptotic competency, we induced aerobic glycolysis in control neurons by exposing the cells to 100 μM cobalt-(II)-chloride (CoCl_2_) and 100 μM deferoxamine (together termed CoDo) (Figure 6H). CoDo provoked a broad hypoxic phenotype in control iNs, which included increased aerobic glycolysis, as evident from a 2.9-fold increase in lactate production and a significantly increased translocation of PKM2 into the nucleus (Figures 6I, 6J, and S6H). To monitor the acute cytonuclear translocation changes of PKM2 in response to CoDo, we cloned an EGFP-tagged PKM2 fusion protein (EGFP:PKM2) into a lentiviral vector and transduced fully converted control iNs to avoid PKM2 effects on the direct conversion process (Figure 6K). Time-lapse fluorescence imaging following 2 days of EGFP::PKM2 expression revealed that CoDo readily induced PKM2 nuclear translocation within an hour of treatment (Figure 6L). Nonetheless, as with AD iNs, we detected no basal toxicity of CoDo at this low concentration (Figures 6M, 6N, and S6G). However, when we exposed CoDo-treated control iNs to 0.3 μM ABT-737, a significant 4.3-fold increase in Casp3-positive iNs was evident (Figures 6M, 6N, S6E, and S6F). However, CoDo also elicited effects that went markedly beyond the phenotypes observed in AD iNs in that it decreased neuronal MMP and metabolic flux into the TCA cycle (Figures S6G and S6I). To more authentically simulate neuronal AD phenotypes in control iNs, we assessed *PKM2*-overexpressing iNs (*PKM2*-OE; Figure 6K). UHPLC-MS metabolomics of *PKM2*-OE iNs revealed a pattern of glycolytic metabolite accumulation very similar to AD iNs, especially for 1,3-BP-glycerate, PEP, and secreted lactate (Figure S6I). Similar to AD iNs, *PKM2*-OE did not affect the ^13^C_6_-glucose flux into mitochondria, but it did not alter the NAD^+^/NADH ratios in control iNs (Figures S4N and S6H–S6J). Importantly, however, *PKM2*-OE alone is sufficient to induce apoptotic competency in healthy iNs, indicating that *PKM2* greatly mediates a concerted metabolic switch and associated cell death vulnerability of old neurons (Figure S6K). Thus, in contrast to other cell types where the Warburg effect is linked to apoptotic escape, neurons respond to the switch toward aerobic glycolysis with an increased competency to functionally respond to an apoptotic stimulus, contributing to exacerbated neuronal cell death in AD.

### Chemical inhibition of PKM2 prevents its nuclear translocation and ameliorates neuronal AD phenotypes

**Figure 7:**
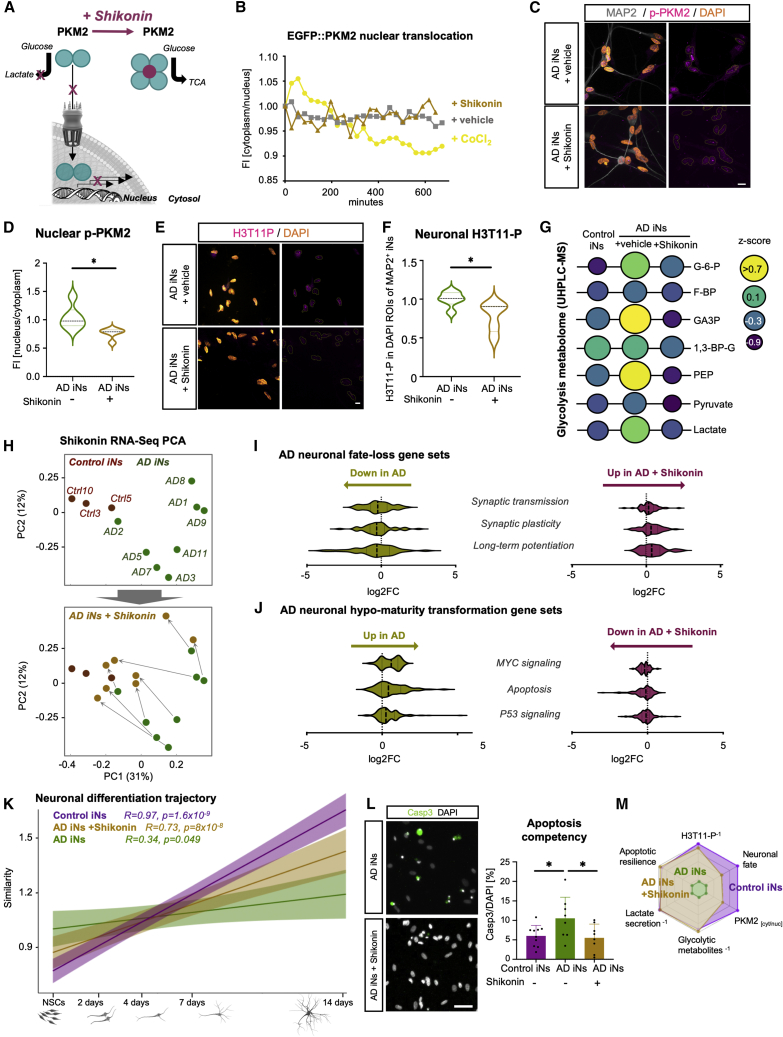
PKM2 inhibition ameliorates PKM2-induced apoptotic competency (A) Schematic: shikonin treatment prevents PKM2 nuclear translocation and increases metabolic enzymatic activity. (B) Longitudinal EGFP::PKM2 localization in vehicle-treated (n = 3), CoCl_2_-treated (n = 4), and CoCl_2_+shikonin-treated (n = 4) control iNs. (C–F) Immunostaining and quantification of nuclear p-PKM2 (C and D) and H3T11-P (E and F) in AD iNs with and without shikonin (vehicle, n = 6; shikonin, n = 5). Scale bars, 10 μm. (G) Glycolytic metabolites measured by UHPLC-MS-based metabolomics. Size and color of the circles are indicative of abundance (control, n = 3; AD, n = 4; AD-shikonin, n = 4). (H) PCA-based bulk RNA-seq (control, n = 3; AD and AD-S, n = 8). (I and J) Transcriptomic analysis of AD neuronal fate-loss gene sets (I) and hypo-maturity gene sets (J) in control (n = 3), AD (n = 8), and AD + shikonin iNs (n = 8) after 10 days of treatment. (K) Similarity profiles of control, AD, AD + shikonin iNs to neuronal differentiation trajectory of neural stem cells to neurons (Schafer et al., 2019). (L) Quantification of immunostainings for cleaved caspase-3/DAPI in control (n = 9), AD (n = 8), and shikonin-treated AD (n = 8) iNs (one-way ANOVA, DF: 25, F = 4.027, p = 0.03). Scale bars, 50 µm. (M) Radar plot of described phenotype and rescue with shikonin. (D–L) Dots represent individual donors throughout the figure. Bars, mean; error bars, SD; violin plots, median and quartiles. Significance: unpaired t test, ^∗^p < 0.05.

While the PKM1 isoform assembles into a catalytically active tetramer, PKM2 lacks this tendency. Instead, it promotes a metabolic switch by (1) accumulating glycolytic metabolites and directing pyruvate toward lactate production and (2) triggering glycolytic gene expression in the nucleus. Given the pivotal role of PKM2 in many cancers, compounds that tetramerize PKM2 have been developed to reduce aerobic glycolysis and prevent its malignant nuclear activity (Figure 7A; Chen et al., 2011; Li et al., 2018; Zhao et al., 2018). Indeed, we observed that 10 μM of the PKM2-inhibitor shikonin efficiently blocked the nuclear translocation of EGFP:PKM2 within hours, as evidenced in live-cell time-lapse fluorescence imaging of control iNs (Figure 7B). Treatment for up to 10 days did not result in any apparent neuronal morphological alterations, and immunocytochemical analysis revealed that prolonged shikonin treatment sustained the significantly reduced nucleocytoplasmic ratios of p-PKM2 by 15% (Figures 7C and 7D). We observed that shikonin treatment further resulted in a 1.4-fold decreased H3T11-P signal in AD iNs at day 10 (Figures 7E and 7F) and led to a 50% reduction in neuronal protein levels of total PKM2 (Figure S7A). Because *PKM2*-OE is sufficient to induce a Warburg-like metabolic switch, we next posited the question as to whether shikonin treatment would ameliorate Warburg-like signatures and restore a mature neuronal metabolic profile. We repeated semiquantitative UHPLC-MS, and, indeed, AD iNs treated with shikonin globally reflected the metabolic landscape of control iNs. No accumulation of upstream PEP or 1,3-BP-glycerate was detected, indicating a full re-gain of regular PKM1 enzymatic activity (Figure 7G). Furthermore, PKM2 tetramerization restored low global levels of glycolytic metabolites (Figure S7B) and normalized neuronal lactate secretion to a basal level (Figure S7C). Shikonin also restored NAD^+^ in AD iNs, without affecting mitochondrial membrane potential or glucose flux into mitochondria (Figures S4O, S7D, and S7E). This finding indicates that the AD phenotype may depend on, but substantially extends from, the age-associated mitochondrial decline observed in old human neurons (Kim et al., 2018). Because shikonin treatment could efficiently block the nuclear translocation of PKM2, we assessed transcriptome-wide effects of shikonin treatment on AD and control iNs (n = 8 shikonin-treated AD iNs, n = 8 vehicle-treated AD iNs, and n = 3 untreated control iNs). The AD iN samples clustered separately from the control samples along PC1/PC2 based on differentially expressed genes, and strikingly, treatment with shikonin markedly and consistently led to a global transcriptome shift of all AD iN samples toward the control samples (Figure 7H). Furthermore, shikonin specifically alleviated cancer-transformation-like hypo-mature transcriptome signatures from the AD iNs, as it removed oncogenic transformation and apoptosis-related gene sets and restored normal gene expression patterns related to mature synaptic properties (Figures 7I and 7J). Partial neuronal fate loss in AD iNs had been reported previously and was also evident from this independent RNA-seq experiment (Figure 7K; Mertens et al., 2021). Importantly, shikonin-treated AD iNs partially reversed this signature and showed increasing mapping with progressive neuronal maturation (Figures 7K and S7F). Because apoptotic competency is characteristic of immature neurons and nuclear PKM2 can drive apoptotic effector gene expression in old AD iNs, we sought to functionally address whether PKM2 tetramerization could indeed prevent neuronal cell death in response to an apoptotic stimulus. We further quantified Casp3-positive neurons following ABT-737 exposure in both the presence and absence of shikonin. We detected a substantial decrease in apoptotic neurons in response to shikonin treatment (Figure 7L). Our data indicate that PKM2 inhibition restored global transcriptomic and functional features of mature neuronal resilience on several levels close to normal (Figure 7M). These findings demonstrate a key role for PKM in controlling the human neuronal metabolic identity and neuronal fitness and resilience and suggest that targeting PKM could positively affect sporadic AD phenotypes in age-equivalent, patient-derived human neurons.

## Discussion

Cellular stress and injury represent well-established triggers of cellular de-differentiation in diverse biological systems (Jopling et al., 2010; Poplawski et al., 2020; Renthal et al., 2020; Tzahor and Poss, 2017), and features of neuronal fate instability and de-differentiation have been described as pathological hallmarks of AD (Arendt, 2012; Herrup and Yang, 2007; McShea et al., 2007; Yang et al., 2001). PM AD single-cell transcriptome analyses have indicated that AD is associated with the evidence of metabolic reprogramming to aerobic glycolysis in neurons (Marinaro et al., 2020). In this context, our current findings suggest that a cancer-like metabolic switch underlies fate instability and several downstream AD features in patient-derived iNs, such as reduced morphological complexity, lower numbers of synaptic-like structures, and reduced frequency of Ca^2+^ neuronal activity (Mertens et al., 2021). This Warburg-effect-like metabolic switch to aerobic glycolysis was predicted by gene expression module-trait-relationship analyses, which distinguished eight candidate genes that appeared to be highly linked to the clinical manifestation of AD in the patient-derived iNs. Isoform switching of *PKM* is characteristic of the Warburg effect and is a prime example of the roles of metabolism beyond the mere adaptations to energy demands (Alves-Filho and Pålsson-McDermott, 2016; Traxler et al., 2021). Several proteome studies of PM brain tissues and CSFs from patients with AD revealed that PKM is a prominent glycolytic enzyme correlating with AD pathology (Higginbotham et al., 2020; Johnson et al., 2020). Here, we show that an isoform switch from PKM1 to PKM2 occurs in AD iNs, leading to pathogenic PKM2 accumulation and activity in the nucleus. PKM2 is known to specifically interact with and enhance the transcription factors STAT3 and HIF1α, which are known to exert stress-related and pro-oncogenic programs (Alves-Filho and Pålsson-McDermott, 2016; Yang et al., 2012a). This is in accordance with previous findings describing an upregulation of oncogenic signaling pathways in AD neurons (iNs and PM brain tissue), including HIF1α signaling that is aberrantly activated despite a normoxic environment (Marinaro et al., 2020; Mertens et al., 2021). Our data further provide evidence that metabolic rewiring via PKM2 is causative for AD-related neuronal defects, as the induction of glycolysis in iNs from healthy age-matched donors increases the susceptibility to apoptotic stimuli. This change necessitates the nuclear translocation of PKM2, and we and others have previously demonstrated that normal human aging leads to an impairment of nucleocytoplasmic transport and compartmentalization (Jovičić et al., 2015; Mertens et al., 2015). Thus, it remains to be determined whether young neurons react similarly or whether this effect is indeed age dependent. Furthermore, the observations of both alterations within metabolic genes in PM AD neurons (Marinaro et al., 2020) and age-related mitochondrial impairment in iNs (Kim et al., 2018) raise the question of whether metabolic rewiring is indeed a pathogenic program in AD or rather an adaptation to mitochondrial dysfunction. Defects in mitochondrial respiration, accumulation of mtDNA damage, impaired mitophagy, or increased ROS production are hallmarks of aging and contribute to the susceptibility of neurons to diseases (Chen et al., 2020; Fang et al., 2019). While mitochondrial dysfunctions are known to contribute to the pathology of AD and have been found in PM brain tissues (Swerdlow, 2011), our data support the view that the activation of this anti-neuronal metabolic state is not merely an adaptation to mitochondrial defects but is deliberately mobilized to support the metabolic needs of the pathogenic program unfolding within AD iNs, which may eventually lead to the mitochondrial pathologies characteristic of AD. The present data indicate that the switch to aerobic glycolysis is independent of age or ApoE genotype, similar to the Warburg effect in cancer, wherein increased aerobic glycolysis is observed despite functional mitochondria (Vaupel and Multhoff, 2021). These findings are based on our model that maintains age-associated features, among others mitochondrial dysfunctions that contribute to the susceptibility of old neurons to diseases (Kim et al., 2018). This age-related mitochondrial dysfunction does not exclude the possibility that AD iNs do not re-wire mitochondrial metabolism to generate precursors for epigenetic modulation (Traxler et al., 2021). Exacerbated mitochondrial dysfunctions might further emerge in the later stages of disease progression.

Extensive research in the cancer fields has well established that successful malignant transformation of a cell necessitates substantial rewiring between metabolic states (Ward and Thompson, 2012). As a result, metabolic regulators such as PKM have emerged as part of the focus of “anti-Warburg” drug development to fight cancer (Chen et al., 2011; Su et al., 2019; Wang et al., 2018; Zhao et al., 2018). Here, we show consistent data in this regard by utilizing aged human neurons in which the chemical inhibition of PKM2 translocation reduced PKM2 loads in the nucleus and restored a mature neuronal metabolic profile. However, in contrast to the data from cancer research, where PKM2 tetramerization induced the apoptosis of tumor cells, in our system, shikonin treatment restored apoptotic brakes that enabled mature neurons to survive for decades (Benn and Woolf, 2004; Kole et al., 2013). These seemingly divergent observations can be explained by the fact that the inhibition of PKM2 in neurons slows the glycolytic rate (Zhao et al., 2018) to prevent its toxic effect on neurons (Zheng et al., 2016) and inhibits the nonmetabolic nuclear roles of PKM2 that cause epigenetic fate loss of neurons. Thus, PKM2 inhibition in neurons not only leads to a decrease in toxic glycolytic metabolites but also reverses the fate loss and thus re-instates neuronal brakes to prevent apoptosis. Further research is needed to understand the similarities and differences between the metabolic switch in aged human neurons and different types of cancer. Importantly, here we have identified PKM2 as a target and shikonin as a lead compound that targets the metabolic AD signature and reduces AD features in human iNs. These findings may have direct clinical relevance, as compounds targeting PKM are currently being assessed in clinical trials but not in the context of neurodegeneration (Li et al., 2018).

Our study offers detailed insights into the metabolic rewiring of age-equivalent neurons from patients with sporadic AD and identifies PKM2 as a key regulator of metabolic and other AD-related changes. These insights contribute toward a better understanding of the age-dependent progression of AD and provide an impetus for the redirection of the existing therapeutic strategies for AD.

### Limitations of study

One limitation of the iN model system is the lack of interaction with surrounding glial cells, which contributes to the metabolic homeostasis in the brain (Afridi et al., 2020). The establishment of powerful tools to reprogram somatic cells and iPSCs into induced astrocytes, oligodendrocytes, and microglia will enable co-culture experiments comprising iNs and other cell types of the brain to study the metabolic interactions during normal aging and neurodegeneration. Furthermore, our system does not recapitulate the effects of long-term exposure to Warburg-like metabolic changes, as recurrent media changes prevent the accumulation of toxic proteins or metabolites that might lead to additional pathologies. Long-term multicellular 3D cultures developed for postmitotic iNs might enhance our understanding of long-term exposure to the metabolic switch in AD.

## STAR★Methods

### Key resources table

**Table:** 

REAGENT or RESOURCE	SOURCE	IDENTIFIER
**Antibodies**		

Mouse anti Beta-III-tubulin	BioLegend	Cat#MMS-435P; RRID:AB_2313773
Rabbit anti Beta-III-tubulin	BioLegend	Cat#802001; RRID:AB_2564645
Mouse anti NeuN	EMD Millipore	Cat#MAB377; RRID:AB_2298772
Rabbit anti NeuN	Cell Signaling	Cat#24307T; RRID:AB_2651140
PE-conjugated anti PSA-NCAM	Miltenyi Biotec	Cat#130-117-394; RRID:AB_2727931
Rabbit anti Synapsin	Merck	Cat#574778; RRID:AB_565174
Mouse anti PSD-95 (clone K28/42)	NeuroMab	Cat#75028; RRID:AB_2292909
Chicken anti Map2	Abcam	Cat#ab5392; RRID:AB_2138153
Rabbit PKM2 (phospho-Ser37)	Sabbiotech	Cat#11456
Mouse PKM2	R&D systems	Cat#MAB72441
Rabbit Histone 3 (phospho T11)	Abcam	Cat#ab5168, RRID:AB_304759
Rabbit cleaved caspase 3 (asp175)(5A1E)	Cell Signaling	Cat#9664; RRID:AB_2070042
Mouse PDH-E1a	Santa Cruz	Cat#sc-377092; RRID:AB_2716767
Mouse PKM2	ThermoFisher Scientific	Cat#TA190266
Alexa Fluor 488-conjugated Donkey Anti Rabbit IgG	Thermo Fisher Scientific	Cat#A-21206; RRID:AB_2535792
Alexa Fluor 647-conjugated Donkey Anti Rabbit IgG	Thermo Fisher Scientific	Cat#A-31573; RRID:AB_2536183
Alexa Fluor 488-conjugated Donkey Anti Mouse IgG	Thermo Fisher Scientific	Cat#A-21202; RRID:AB_141607
Alexa Fluor 647-conjugated Donkey Anti Mouse IgG	Thermo Fisher Scientific	Cat#A-31571; RRID:AB_162542
Alexa Fluor 647-conjugated Donkey Anti Chicken IgY	Millipore	Cat#AP194SA6; RRID:AB_2650475
Cy3-conjugated Donkey Anti-Mouse IgG	Jackson ImmunoRes.	Cat#715-165-151; RRID:AB_2315777
Cy3-conjugated Donkey Anti-Rabbit IgG	Jackson ImmunoRes.	Cat#711-165-152; RRID:AB_2307443
Anti-Mouse HRP Detection Module	Bio-Techne	Cat#DM-002

**Bacterial and virus strains**		

NEB Stable Competent E. coli	New England Biolabs	Cat#C3040H

**Biological samples**		

Fibroblast cultures from cohort (see Figure S1A)	this study and Mertens et al. (2021)	Figure S1A

**Chemicals, peptides, and recombinant proteins**		

Trizol-LS reagent	Thermo Fisher	Cat#10296010
Puromycin	Sigma Aldrich	Cat#P8833
TrypLE dissociation reagent	Thermo Fisher Scientific	Cat#12604013
B-27 supplement	Thermo Fisher Scientific	Cat#17504044
N2 supplement	Thermo Fisher Scientific	Cat#17502048
Non-Essential Amino Acids (NEAA) supplement	Thermo Fisher Scientific	Cat#M7145
Laminin coating reagent	Sigma Aldrich	Cat#L2020
Geltrex coating reagent	Thermo Fisher Scientific	Cat#A1413201
Y-27632 (ROCK inhibitor)	StemCell Technologies	Cat#72308
Dibutyryl-cyclic-AMP	Santa Cruz	Cat#sc-201567B
Recombinant Noggin	R&D systems	Cat#6057NG
CHIR99021	LC Laboratories	Cat#C-6556
LDN-193189	Sanova Pharma	Cat#HY-12071
A83-1	Santa Cruz Biotech	Cat#K1119
Forskolin	LC Laboratories	Cat#F-9929
SB-431542	MedChem	Cat#HY-10431
Poly-L-ornithine coating reagent	Sigma Aldrich	Cat#A-004-C
Doxycycline	Sigma Aldrich	Cat#089M4004V
KnockOut Serum Replacement (KOSR)	Thermo Fisher Scientific	Cat#10828010
BrainPhys culture media	StemCell Technologies	Cat#05790
Shikonin	Santa Cruz	Cat#sc-200391
D-Glucose C13	Santa Cruz	Cat#sc-239643A
Cobaltchloride	Santa Cruz	Cat#sc-252623
Deferoxamine-mesylate	Sigma Aldrich	Cat#138-14-7
ABT737	Tocris	Cat#6835
JC-1 dye	Thermo Fisher	Cat#T3168
Triton X-100 reagent	Sigma Aldrich	Cat#X100
DAPI fluorescence reagent for DNA	Sigma Aldrich	Cat#D8417
TURBO DNAse for NGS library preparation	Ambion	Cat#AM2238
DNAse for cell culture	Roche	Cat#4716728001
TruSeq Stranded mRNA Sample Prep kit	Illumina	Cat#20020594
SuperScript III First-Strand Synthesis System	Thermo Fisher Scientific	Cat#18080051
Tagment DNA Enzyme and Buffer kit	Illumina	Cat# 20034197
cOmplete EDTA-free Protease Inhibitor Cocktail	Roche	Cat#11836170001
PhosSTOP Phosphatase Inhibitors	Zymo Research	Cat#PHOSS-RO
RIPA Lysis and Extraction Buffer	Thermo Fisher Scientific	Cat#89900

**Critical commercial assays**		

Lactate assay kit	Merck	Cat#MAK064-KT
Lactate assay kit	Biocat	Cat#K607
ATP/ADP ratio kit	Sigma Aldrich	Cat#MAK135
NAD/NADH Kit	Sigma Aldrich	Cat#MAK037
NAD-NADH-Glo Assat	Promega	Cat#G9071
Caspase-Glo 3/7	Promega	Cat$G8090
PKM activity assay	Sigma Aldrich	Cat#MAK072
ProteinSimple 12-230 kDa Separation Module	Bio-Techne	Cat#SM-W004
ProteinSimple Jess 25-Capillary Cartridges	Bio-Techne	Cat#PS-CC01

**Deposited data**		

RNA-Seq after shikonin treatment	ArrayExpress	E-MTAB-11855
UHPLC-MS Metabolomics	Metabolomics Workbench	ST002213
UHPLC-MS Metabolomics Shikonin	Metabolomics Workbench	ST002214

**Oligonucleotides**		

TruSeq RNA-Seq Single Indexes Set A	Illumina	Cat#20020492
TruSeq RNA-Seq Single Indexes Set A	Illumina	Cat#20020493

**Recombinant DNA**		

pLVXUbC-rtTA-Ngn2:2A:Ascl1	Herdy et al., 2019	Addgene #127289
pCSC-hSyn1::dsRed	Mertens et al., 2015	N/A
pLVXTP-EGFP-PKM2	This paper	N/A

**Software and algorithms**		

STAR Aligner	https://github.com/alexdobin/STAR	N/A
Babraham Bioinformatics TrimGalore	https://www.bioinformatics.babraham.ac.uk/projects/trim_galore/	N/A
Samtools	https://samtools.github.io/	N/A
DESeq2	https://github.com/mikelove/DESeq2	N/A
Hypergeometric Optimization of Motif Enrichment (HOMER)	http://homer.ucsd.edu/homer/	N/A
deepTools	https://deeptools.readthedocs.io/	N/A
bedtools	https://bedtools.readthedocs.io/	N/A
MBF Bioscience Neurolucida	https://www.mbfbioscience.com/neurolucida	N/A
Integrative Genomics Viewer - Broad Institute (IGV)	https://software.broadinstitute.org/software/igv/	N/A
Metaboanalyst	https://www.metaboanalyst.ca/	N/A
FlowJo	https://www.flowjo.com/	N/A
GraphPad Prism	https://www.graphpad.com/	N/A
STRING-db	https://string-db.org/	N/A
DAVID Functional Annotation	https://david.ncifcrf.gov/	N/A
Reduce + Visualize Gene Ontology (REVIGO)	http://revigo.irb.hr/	N/A
FACSChorus Software	Becton Dickinson	N/A
ZEN Imaging Software	Carl Zeiss	N/A
ImageJ	https://imagej.nih.gov/ij/	N/A

### Resource availability

#### Lead contact

Further information and requests for resources and reagents should be directed to and will be fulfilled by the lead contact, Jerome Mertens (Jerome.Mertens@uibk.ac.at).

#### Materials availability

This study did not generate new unique reagents.

### Experimental model and subject details

#### Fibroblasts and iNs

Human fibroblasts were obtained from the UCSD Shiley-Marcos Alzheimer‘s disease research center (ADRC) and provided written informed consent. All procedures were approved by local human subjects committees. The cohort consists of 14 male and 8 female donors between 56 and 88 years (Figure S1A), most of whom underwent clinical assessment as part of the UCSD ADRC study. Fibroblasts were cultured in DMEM containing 15 % fetal bovine serum and 0.1 % non-essential amino acids at 37°C with 5 % CO_2_. They were transduced with a lentivirus pLVXUbC-rtTA-Ngn2:2A:Ascl1 (Addgene: #127289) and selected with puromycin (1 μg/ml) as previously described (Mertens et al., 2021). To initiate conversion, puromycin-selected fibroblasts were pooled 3:1 and, after 24 hours, medium was changed to neural conversion medium for three weeks. Neural conversion medium is based on DMEM:F12 and Neurobasal (1:1), supplemented with N2 supplement (1x, ThermoFisher), B27 supplement (1x, ThermoFisher), doxycycline (2 μg/ml, Sigma Aldrich), Laminin (1 μg/ml, Sigma Aldrich), dibutyryl-cyclic-AMP (100 μg/ml, Santa Cruz), human recombinant noggin (150 ng/ml, R&D), LDN-193189 (0.5 μM, Sanova Pharma), A83-1 (0.5 μM, Santa Cruz), CHIR99021 (3 μM, LC Laboratories), forskolin (5 μM, LC Laboratories) and SB-431542 (10 μM, MedChem).

After three weeks of conversion, cells were detached with TrypLe (ThermoFisher) and either plated on Geltrex-coated (ThermoFisher) μ-slides (ibidi) or FACS sorted. For sorting, cells were detached with TrypLE and stained with PSA-NCAM-PE (Milteny Biotec) in sorting buffer (150 mM myo-inositol and 5 mg/mL polyvinyl alcohol in PBS and ddH2O) containing 5 % FBS. Cells were sorted in PBS containing EDTA (Invitrogen), Rock-inhibitor (10 μM), and DAPI and plated in conversion media containing Rock-inhibitor and z-VAD(OMe)-FMK. Cells were treated with shikonin (10 μM, ChemCruz) or NR (300 μM, ChromaDeX) 1 week before FACS sorting until harvest. Cobalt-II-Chloride (100 μM, Santa Cruz) and deferoxamine (100 μM, Sigma Aldrich) treatment was initiated after FACS sorting until harvest.

### Method details

#### Immunocytochemistry

Cells were fixed with 4 % paraformaldehyde and stained in PBS containing 0.05 % Triton-X100 and 5 % FBS. Cells were incubated with primary antibodies PKM2 (Origene, 1:500, TA347018), p-PKM2 (Eubio, 1:500, #11456-2), H3T11-P (Abcam, 1:100, ab5168), cleaved caspase 3 (Cell Signaling, 1:1000, #9664), β-III-tubulin (BioLegend, 1:1000, #802001), Synapsin (Merck, 1:750, #574778), PSD95 (ThermoFisher, 1:300, MA1046), NeuN (CellSignaling, 1:100, #24307T) at 4°C overnight and incubated with secondary antibodies for two hours at room temperature, followed by 10 minutes of DNA staining with DAPI (ThermoFisher, 300nM, D21490). Images were taken with the Leica DMi8 microscope and analyzed in FIJI. Nuclear expression of p-PKM2 was measured as IntDen in region-of-interests (ROIs) set based on DAPI; total neuronal expression was measured as IntDen in ROIs based on MAP2. For assessment of synapse-like structures, neurons were transduced with synapsin-RFP (Addgene #22909). Neuronal morphology was assessed based on β-tubulin using the Neuroanatomy SNT plugin in ImageJ, measuring neurite length from the cell body to the furthest connection, and the complexity of the branching on these neurites.

#### Immunohistochemistry of post-mortem brain sections

Formalin-fixed prefrontal cortex slices embedded in paraffin of 10 healthy old control subjects (Braak 1-2) and 10 patients with sporadic AD (Braak 3-4) were obtained from the Shiley Marcos Alzheimer’s Disease Research Center in San Diego. After deparaffinization, antigen retrieval was achieved using HIER buffer (ThermoFisher) before staining with PKM2 (ThermoFisher, TA190266) and NeuN (Abcam, ab104224) at the suggested dilutions. Background fluorescence was reduced using Sudan Black B before mounting in PVA-DAPCO. Images were taken at the Olympus VS120 automated slide scanner and analyzed using ImageJ. Total PKM2 levels were measured in outer cortical layers, followed by measurement of PKM2 fluorescence intensity in NeuN-ROIs and extended NeuN-ROIs to measure perinuclear regions.

#### Mass spectrometry metabolomics

A total of 150,000 FACS-sorted iNs were treated with ^13^C_6_-Glucose (Santa Cruz, sc-239643A) for six hours before collecting cell pellets and supernatant to resuspend in Lysis Buffer (50:30:20 MeOH:Acetonitrile(ACN):H_2_O, 2 Million cells per mL) or mix 1:25 in Lysis Buffer, respectively. After vortexing for 30 minutes at 4°C, proteins were precipitated when centrifuging for 10 minutes at 18.000 x g at 4°C. The supernatant containing metabolites was resolved over the Kinetex C18 column (2.1 x 150 mm, 1.7 μm, Phenomenex) using a Vanquish UHPLC system and analyzed with the high-resolution Q Exactive mass spectrometer (Thermo Scientific) at 35 °C. A volume of 10 μl for pellets and 20 μl for supernatant analysis was injected for positive and negative ion mode, using a 5 minutes gradient at 450 μl/min from 5% to 95% of ACN/0.1% Formic Acid In Water/0.1% Formic Acid (positive mode) and 95% ACN/5% water/1mM ammonium acetate in 5% ACN/95% water/1mM ammonium acetate (negative mode). Raw files were converted to mzXML file format using Raw converter (He et al., 2015) and technical replicates were used to control technical variability. Only metabolites with a coefficient of variation (CV = SD/mean) < 20% were considered for this report. Metabolite assignment to KEGG compounds was performed using MAVEN, and normalization to protein content measured with nanodrop was performed with Metaboanalyst (Pang et al., 2021). Statistical z-scores were calculated for visualization. Relative glucose consumption was measured by calculating the presence of glucose in the supernatant after six hours of incubation, and as the sum of all detected labeled metabolites in the flux analysis. Metaboanalyst software was used for enrichment analysis of PCs.

#### Targeted metabolic assays

For metabolic assays, fibroblasts were converted for three weeks and 50.000- 100.000 FACS-sorted iNs were plated on Geltrex-coated, white- or black-walled 96-well plates. Four to seven days after re-plating, supernatant was analyzed using colorimetric lactate assays (Biovision, #K607 or Merck MAK064). Attached cells were processed using the Pyruvate Kinase activity assay Kit (Sigma Aldrich, MAK072), the ATP/ADP ratio kit (Sigma Aldrich, MAK135), or the NAD/NADH kit (Sigma Aldrich, MAK037 or Promega, G9071) according to the manufacturers’ instructions. Recording of signals was performed using the Enspire Multimode Plate Reader platform (PerkinElmer).

#### Mitochondrial membrane potential

Three week-converted iNs were treated were stained in suspension using 2 μM of the JC-1 dye (ThermoFisher, T3168) for 20 minutes and analyzed by flow cytometry. Neurons were identified according to PSA-NCAM staining (BD FACS Melody). Analysis was performed using FlowJo v10 (BD Biosciences) in the PSA-NCAM^+^ population. Histograms of red and green fluorescence were assessed, and geometric means were extracted.

#### Capillary Western blot analysis

PDH protein was quantified in lysed pellets of FACS-purified iNs using the ProteinSimple Jess (Biotechne) and the 12-230 kDa Jess Separation Module in the NIR channel. Pellets were lysed in RIPA Lysis and extraction Buffer (ThermoFisher) containing cOmplete EDTA-free Protease Inhibitor Cocktail (Merck, 11873580001) and PhosSTOP (Merck, 4906845001). Anti-PDH antibody (Santa Cruz, sc-377092, 1:50) was incubated for 60 minutes, followed by standard default run settings provided by ProteinSimple. Data analysis was performed using Compass software.

#### Apoptosis assay

To evaluate neuronal resilience, we converted fibroblasts for three weeks and plated two wells of 50.000 FACS-sorted iNs for each donor on Geltrex-coated μ-Plates with 96 wells and black walls (ibidi). Two to five days after sorting, we treated one well of each donor with the Bcl-2 inhibitor ABT-737 (Tocris, 0.16 μM, #6835) for eight hours and fixed all the cells with 4 % paraformaldehyde. Subsequently, we stained for cleaved caspase 3 and either β-III-tubulin or MAP2 (Neuromics, CH22103) and stained DNA with DAPI.

#### Fluorescent cell time-lapse imaging

For time-lapse imaging, fibroblasts were converted for at least three weeks and plated on μ-slide eight-well coverslips. Within one week, cells were transduced with pLVXTP-EGFP-PKM2 according to the Lenti-X GoStick value (Takara). Green fluorescent cells could be observed 24 hours after transduction. To image EGFP::PKM2 localization, we stained the nucleus with siRDNA (Spirochrome) 30 minutes before imaging with the Leica DMi8 microscope in an environmental chamber, allowing cells to be under stable conditions of 37°C and 5% Co2.

#### Electrophysiological analysis

For electrophysiological recordings, three-week-old induced neurons were plated on Geltrex-coated plastic coverslips (Thermanox) and cultured for at least one week after re-plating. Spontaneous synaptic activity and evoked responses were recorded in whole cell patch clamp conditions at room temperature using a Multiclamp 700B amplifier (Molecular Devices) and acquired with DASYLab v.11 (National Instruments) at 20kHz. Patch pipettes with input resistances of 6–8 MΩ were pulled from standard wall glass of 1.5-mm OD (Precision Instruments) and filled with a solution containing (in mM) K-gluconate 100, KCl 10, KOH 10, MgCl2 2, NaCl 2, HEPES 10, EGTA 0.2, D-glucose 5; pH set to 7.3. The bath solution (artificial cerebrospinal fluid) was composed of NaCl 140, KCl 5, CaCl2 2, MgCl2 1, HEPES 5 and D-glucose 10; pH set to 7.5. To record voltage responses of the identified iNs, we used incremental levels of constant, rectangular current steps of 350-ms duration. The initial current step level was –50 to –100 pA, depending on the observed input resistance of the cell. Steps were incremented by + 2.5 pA in successive cycles of stimulation at a rate of 1 Hz. Analysis of the evoked responses was performed in software developed by A. Szücs (NeuroExpress). For each cell, several physiological parameters, including the resting membrane potential, rheobase, input resistance, membrane time constant, and spike amplitude, were measured.

#### WGCNA of RNA-Seq data

WGCNA was performed separately on the transcriptomic data of E-MTAB-10352 and the GSE5281 dataset, using the R package developed by P. Langfelder and S. Horvath (Langfelder and Horvath, 2008). For each dataset, a soft threshold was applied according to the approximate scale-free topology, and the modules were identified by the constructed unsigned gene network. Eigengenes of each module were correlated to the traits of interest (Alzheimer’s yes(1)/no(0), MMSE 1-30, female(0)/male(1), age 53-89, ApoE genotype (23,33,43,34)). Genes of modules significantly correlated to AD and MMSE were extracted and used for gene set enrichment analysis. We summarized all KEGG and UniProt terms for each significant module using GoPlot analysis and plotted them according to their adjusted p-value and non-statistical p-value, as calculated by the R package [z-score = (upregulated – downregulated genes of each term)/root(number of genes in this term)]. We quantified the abundance of each gene in the top 10 KEGG and UniProt terms to check for the most abundant genes and depictured them in a chordplot with the GoPlot package.

#### Integrated Molecular Pathway Level Analysis

For integrative analysis of transcriptomic and metabolomic data, we uploaded transcriptomic data of the E-MTAB-10352 dataset and generated UHPLC-MS metabolomics data of the same patients to the IMPaLa webtool (Kamburov et al., 2011). IMPala generates enrichment analysis and calculates adjusted p-values and rich factors (the degree of enrichment for each term) for genes and metabolites separately and jointly. For analysis, we focused on KEGG pathways and depictured the top KEGG pathways according to the joint adjusted p-value in a bubble plot using R.

#### ATAC-Seq analysis

To analyze the chromatin openness around genes regulated by PKM-regulated transcription factors, we downloaded human ChIP-Seq data from ReMap2020 (Chèneby et al., 2018), and using deeptools we calculated chromatin openness according to the ATAC-Seq dataset E-MTAB-10352 from the patients mentioned in this study (Ramírez et al., 2016). Heatmaps of open chromatin around the transcriptional start site (-1500, +2500) and text files of the respective profiles were generated for each patient with deeptools. We subsequently analyzed the mean of the profile openness of each patient with Prism to perform statistical analysis. Based on the ReMap2020 genes, we extracted mRNA abundance from fpkm-normalized transcriptomic data and calculated if the respective genes were up- or downregulated in AD; then we performed gene set enrichment analysis on the upregulated genes. Additionally, we performed HOMER motif finding for DE of ATAC peaks identified with HOMER, as previously described (Mertens et al., 2021).

#### mRNA sequencing (RNA-Seq) analysis

iNs were directly sorted into Trizol LS reagent (Thermo Fisher) and RNA was extracted according to the manufacturer’s protocol, followed by TURBO DNase digestion (Thermo Fisher). RNA integrity was assessed using the Bioanalyzer High Sensitivity RNA Analysis Kit (Agilent). A cDNA library was generated using the TruSeq Stranded mRNA Sample Preparation Kit (Illumina) and sequenced paired-end 75 at the NExtSeq 500 platform. Read trimming was performed using TrimGalore and mapped with STAR to the hg38 before generating rawcounts using featureCounts. Differential expression analysis was performed after variance stabilizing transformation (vst) using DESeq2. Hypomaturity and differentiation trajectory gene sets were extracted from Mertens et al. (2021) and log_2_ fold changes for each gene were plotted. Pearson correlation between our iN transcriptomic dataset and published iPSC-neural stem cell (NSC) differentiation data (Schafer et al., 2019) was performed in R based on fpkm-normalized counts. To analyze *PKM* splicing in paired-end transcriptomic data, we BAM files of each patient were assessed with the Integrative Genomics Viewer (IGV) and Exon-8-to-9, and Exon-9-to-10 splicing was quantified using Sashimi plot function. Splicing changes were further confirmed with HOMER exon counts, which was also applied to the transcriptomic dataset of NSC differentiation.

#### Seahorse mitochondrial analysis

Six week-converted iNs were seeded at equal concentrations on a Seahorse XF96 Microplate coated with Geltrex and incubated for 48 hours to allow cells to adhere. Medium was then changed to Seahorse phenol red-free DMEM supplemented with N2 and B27 to perform the XF Cell Mito stress test (Agilent) according to the manufacturer’s instructions.

### Quantification and statistical analysis

Log_2_ fold changes of pseudobulk analysis of single-cell transcriptomics of human post-mortem brain tissues were extracted from a published dataset (Grubman et al., 2019) for PKM2-related genes and plotted using R for each described cell type. ImageJ was used to analyze immunofluorescence images, and the detailed procedure is described in the ICC section. Prism was used to calculate statistics for non-omics data and normal distribution was evaluated using the Shapiro-Wilk test. Data was analyzed with the method indicated in each figure. Significance evaluations are marked as ^∗^p<0.05, ^∗∗^p<0.01, ^∗∗∗^p<0.001 in the figures and the statistical test including n numbers are included in the figure legends. Metabolomics data were normalized to cell count and statistics were performed using the normalization to median in Metaboanalyst software.

## Data Availability

• iN RNA-Seq and ATAC-Seq data have been deposited on ArrayExpress and are publicly available as of the date of publication. UHPLC-MS metabolomics data were uploaded on Metabolomics Workbench. Post-mortem transcriptome data were processed from previously published data available on GEO. Uncropped images and all values from the graphs are available in Data S1. • This paper does not report original code. • Any additional information required to reanalyze the data reported in this paper is available from the lead contact upon request.

## Supplemental information

Document S1. Figures S1–S7

Data S1. Unprocessed data underlying the display items in the manuscript Provides uncropped images of immunofluorescence analyses related to Figures 1D, 6A, 6E, 6K, 6M, 7L, and S1B and uncropped blots of Simple western Blot analysis related to Figure S4D. Additionally, unprocessed data related to all main and supplemental Figures are provided.

Document S2. Article plus supplemental information
