# Supplementary material for: Warburg-like metabolic transformation underlies neuronal degeneration in sporadic Alzheimer’s disease
Source: Cell Metab. 2022 Sep 6;34(9):1248–1263.e6. doi: 10.1016/j.cmet.2022.07.014 (PMC9458870; doi:10.1016/j.cmet.2022.07.014)
Supplement: Document S1. Figures S1–S7 [file mmc1.pdf]

Cell Metabolism, Volume *34*

Supplemental information

Warburg-like metabolic transformation underlies neuronal degeneration in sporadic Alzheimer's disease

Larissa Traxler, Joseph R. Herdy, Davide Stefanoni, Sophie Eichhorner, Silvia Pelucchi, Attila Szücs, Alice Santagostino, Yongsung Kim, Ravi K. Agarwal, Johannes C.M. Schlachetzki, Christopher K. Glass, Jessica Lagerwall, Douglas Galasko, Fred H. Gage, Angelo D'Alessandro, and Jerome Mertens

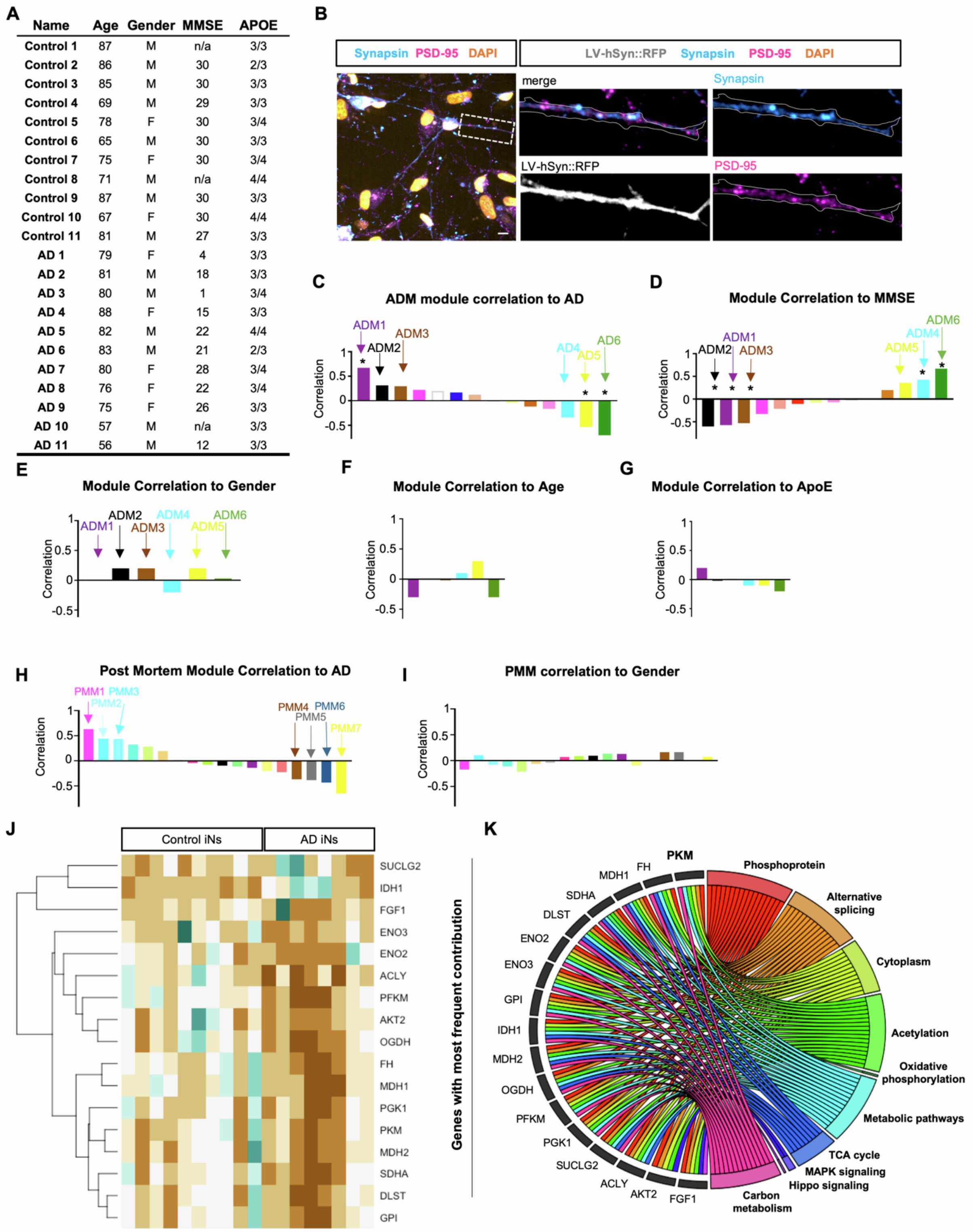

Figure S1. Gene network analysis of a cohort of control and AD iNs

Related to Figure 2. (**A**) Clinical data of 11 control and 11 patients with AD in this study. **(B)** iNs labeled with lentivirus expressing RFP under synapsin promoter, stained for synapsin and PSD95. Scale bar= 10 µm **(C-D)** Gene modules of iNs identified by WGCNA and their correlation to AD and MMSE. **(E-G)** Correlation of ADM1-ADM6 to gender, age and ApoE. **(H-I)** Gene modules of post-mortem brain and their correlation to AD and the only known variable, gender. **(J)** Chordplot of the 17 most abundant genes in the top 20 KEGG pathways and UniProt keywords and their pathway contribution. **(K)** Heatmap of gene expression of the 17 most abundant genes across the iN cohort.

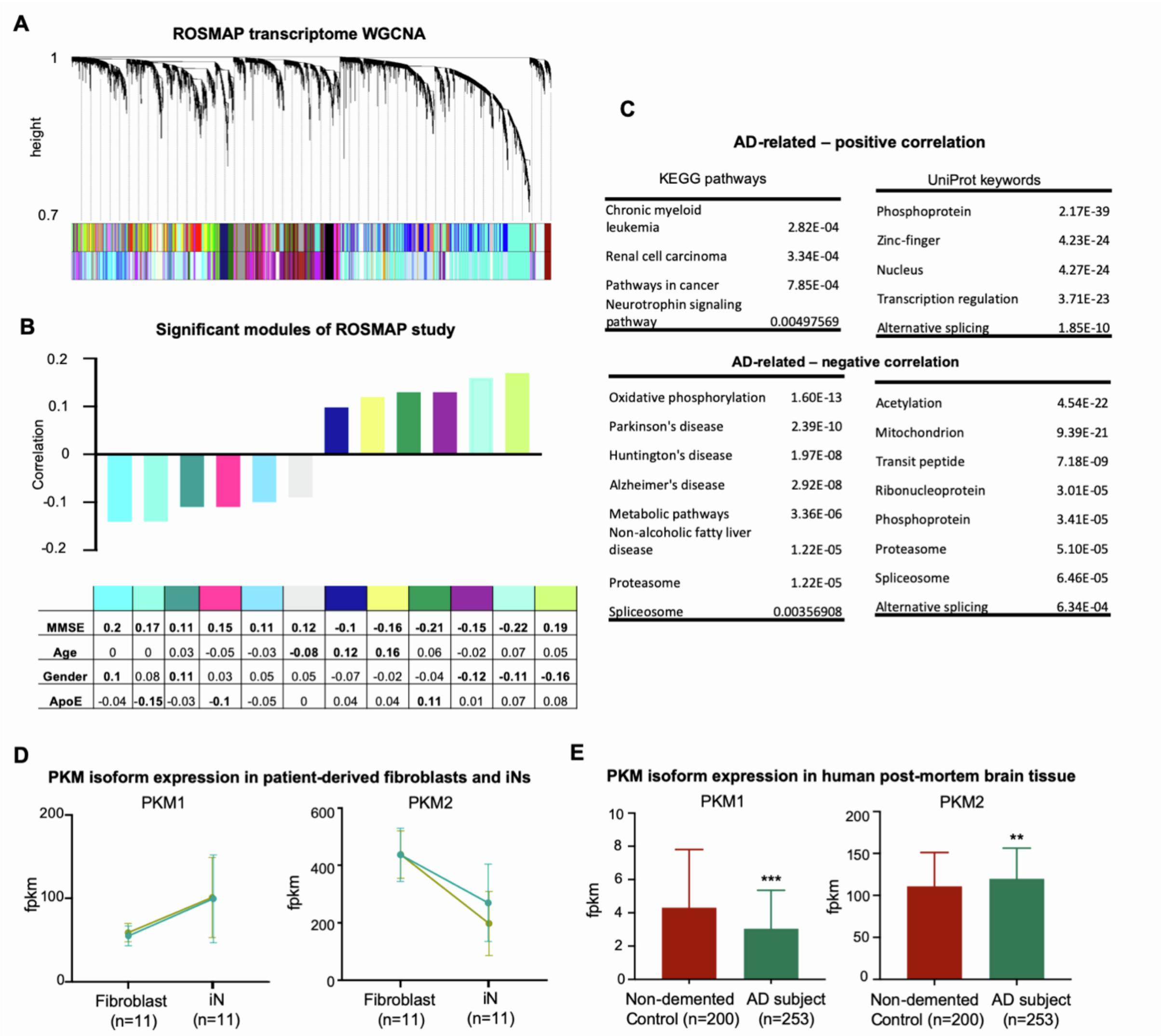

Figure S2. Gene network analysis of post-mortem brains of ROSMAP study

Related to Figures 2 and 3. **(A)** Cluster dendrogram of WGCNA analysis of vst-normalized ROSMAP transcriptomic data. **(B)** Gene modules significantly correlated to AD as a bar plot and their correlation values presented in the table. **(C)** KEGG pathways and UniProt keywords of AD-related gene modules. **(D)** PKM1 and PKM2 isoform counts comparing starting fibroblasts and iNs from the same donors. **(E)** FPKM-normalized PKM1 and PKM2 isoform counts in ROSMAP based on post-mortem diagnosis (n=633). Significance: Mann-Whitney test, **p=0.005, ***p=0.0005.

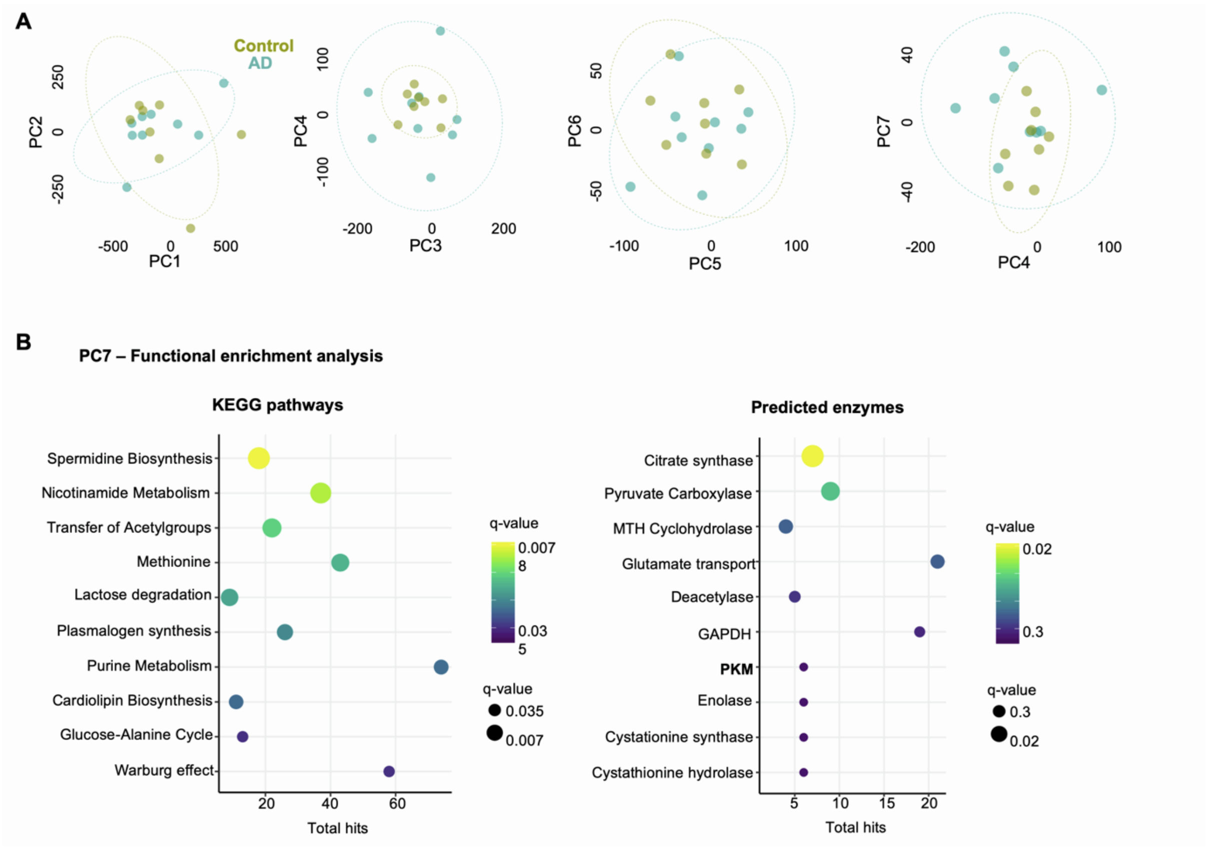

Figure S3. PCA of UHPLC-MS metabolomics in iNs

Related to Figure 4. **(A)** Dotplots of PC1-PC7 comparing control and AD iNs based on their metabolic profile (n=10 per group). **(B)** Enrichment analysis using Metaboanalyst of top 20 metabolites of PC7.

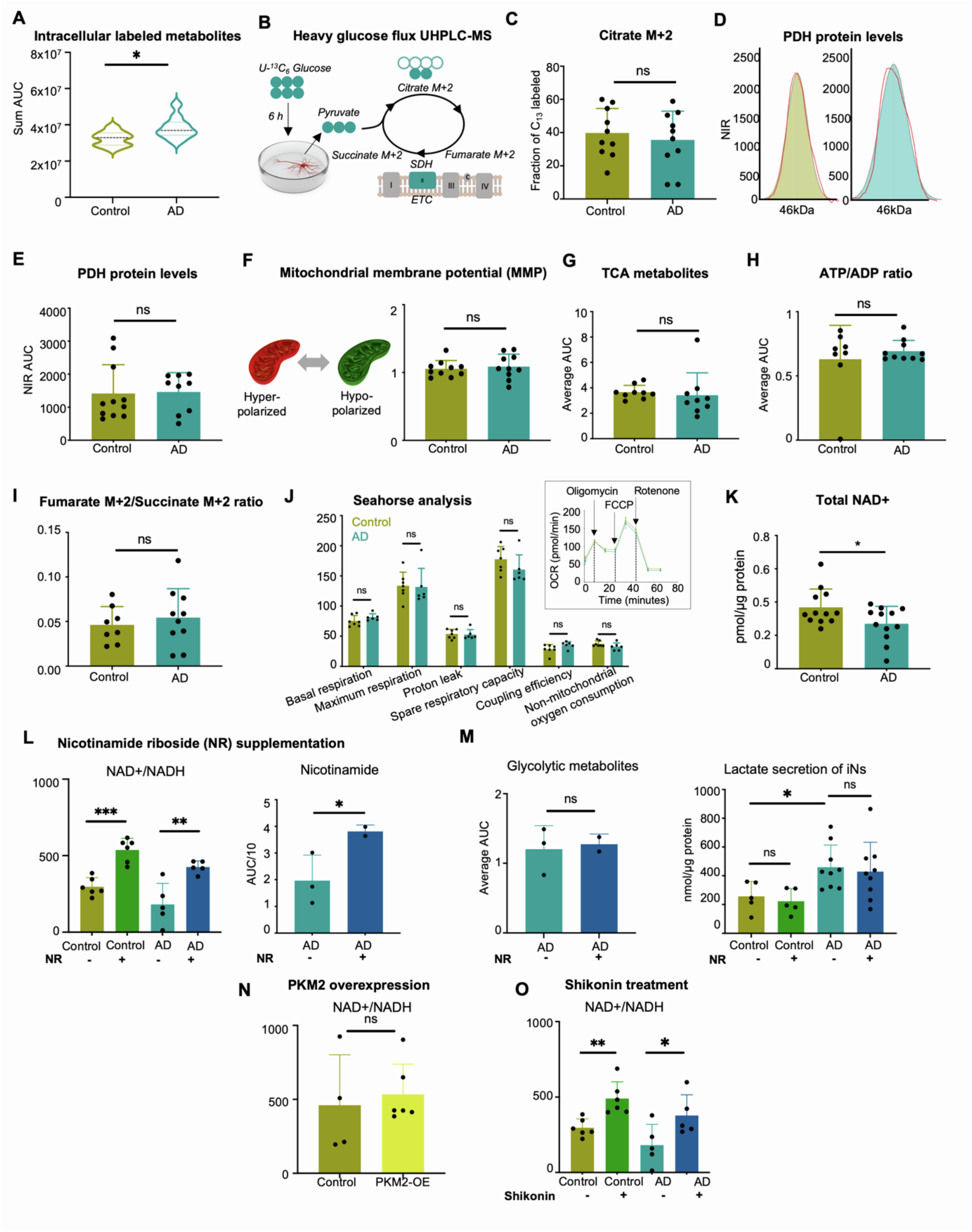

Figure S4. Mitochondrial function remains unaffected in AD iNs

Related to Figure 4. **(A)** Glucose consumption as sum of labeled metabolic intermediates after six hours of treatment with $U\text{-}^{13}C_6$-Glucose. **(B)** Scheme of glucose flux to TCA cycle and involvement in electron transport chain (ETC). **(C)** Fraction of heavy-labeled Citrate. **(D-E)** Protein level of PDH measured by ProteinSimple (n=11 control, n=9 AD). **(F)** Mitochondrial membrane potential (JC-1 dye) analyzed by FACS (control n=9, AD n=10). **(G)** Average of TCA metabolites in AD iNs (n=9) compared to controls (n=9). **(H)** Ratio of ATP and ADP levels as measured by UHPLC-MS. **(I)** Heavy-labeled fumarate/succinate level (succinate dehydrogenase (SDH) activity). **(J)** Mitochondrial respiration measured by seahorse mitochondrial stress test (n=7 control, n=6 AD). **(K)** Colorimetric assay measuring total free NAD^+ levels. **(L)** Restoration of NAD^+/NADH ratio in control and AD iNs measured by a luminescence assay (n=6 control, 5 AD; one-way ANOVA, DF: 21; F-value 18.31, p-value <0.0001), and the precursor nicotinamide measured by UHPLC-MS (n=3 control, 2 AD) after NR supplementation (300 µM, 72 hours). **(M)** Effects of NR supplementation on lactate secretion (colorimetric assay, n=5 control, n=9 AD, average for each donor of four independent experiments,

one-way ANOVA, DF: 27, F-value 3.677, p-value 0.02) and total glycolytic metabolite levels (UHPLC-MS, n=3 AD, n=2 AD + NR). **(N-O)** Effects of PKM2 overexpression (n=4 control, n=6 overexpression, unpaired t-test) and shikonin treatment (n=6 control, n=5 AD, one-way ANOVA, p-value 0.0021, F-value 7.335, DF 21) on total NAD^+ levels and NAD^+/NADH ratios measured by UHPLC-MS after 10 days of treatment and FACS-purification. Significance: unpaired t-test, *$p<0.05$, **$p<0.001$, ***$p<0.005$.

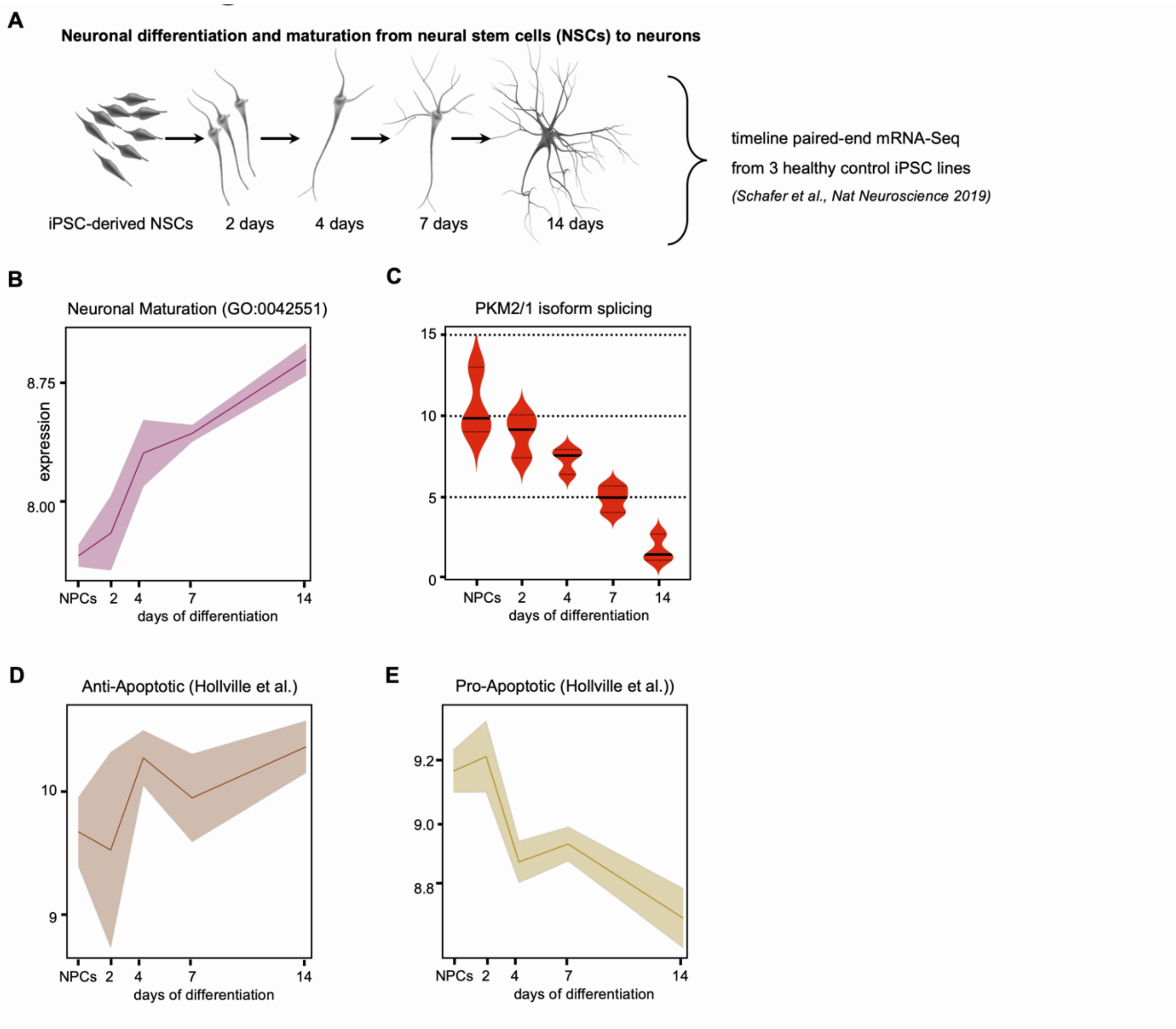

Figure S5. Apoptotic and PKM gene expression during neuronal differentiation

Related to Figure 6**. (A)** Schematic: timeline RNA-Seq differentiating iPSC-derived NSCs (Schafer et al., 2019). **(B)** Genes associated with the GO term "neuronal maturation" (GO:0042551) increase their expression along differentiation. **(C)** PKM2/PKM1 ratio decreases as neurons differentiate. **(D-E)** Differentiating neurons increase gene expression related to the neuronal apoptotic brake and decrease neuronal pro-apoptotic genes, as identified according to Holville et al. (2019).

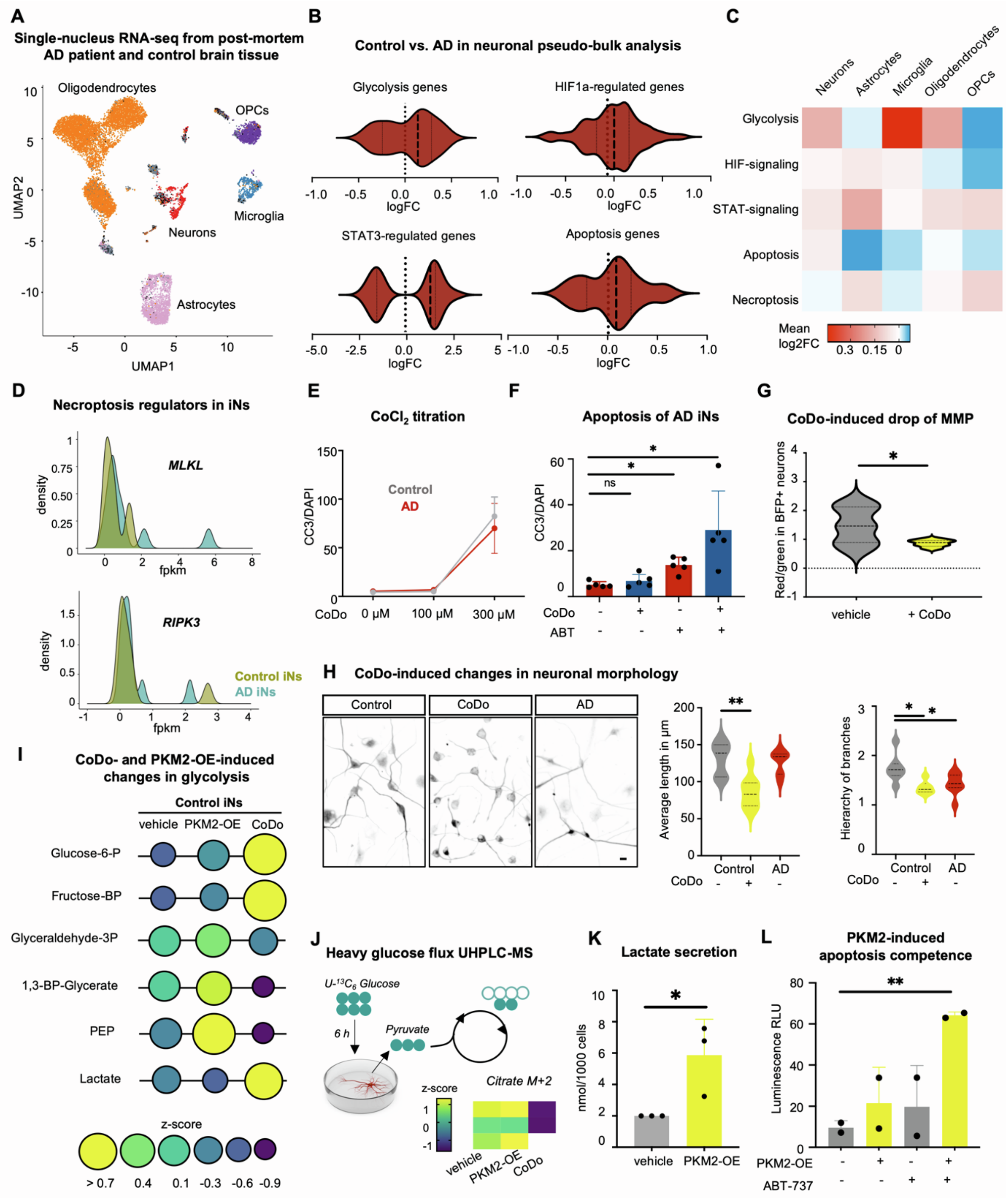

Figure S6. Induction of aerobic glycolysis in AD iNs using CoDo and PKM2 overexpression

Related to Figure 6. **(A)** UMAP of single-nuclei transcriptomic data of post-mortem brain tissue, modified from Grubman et al. (2019). **(B)** Violin plots representing log2FC of PKM-related gene expression in the neuronal subpopulation, and **(C)** heatmap showing median of log2FC of genes related to the indicated pathways comparing different cell populations in the human brain. **(D)** Distribution of FPKM-normalized counts of main necroptosis-related genes in control and AD iNs (n=10 per group). **(E)** Cell death of control and AD iNs with vehicle treatment, 100 µM and 300 µM $CoCl_2$. **(F)** Cell death assessed by cleaved caspase3/β-tubulin-positive cells of AD iNs. **(G)** Mitochondrial membrane potential in control and AD iNs with and without CoDo treatment (n=3 per group). **(H)** Neuronal morphology assessed by β-tubulin staining in control, CoDo-treated control, and AD iNs (n=9, scale bar 10 µm). Average length measured from cell body to end of longest dendrites; hierarchy representing complexity of branching (one-way ANOVA, length: p-value <0.0001, F-value 14.27, DF 26; hierarchy: p-value 0.0089, F-value 6.12, DF 21). **(I)** UHPLC-MS metabolomics comparing control iNs overexpressing GFP::PKM2 for 48 hours (n=3), and CoDo

treated iNs (n=2) compared to GFP-vehicle treated control iNs (n=3). Size and color of circles represent z-scores of respective metabolites. **(J)** UHPLC-MS measurement of labeled citrate after six hours of $^{13}C_6$ Glucose treatment. **(K)** Lactate secretion measured by colorimetric assay in control iNs overexpressing GFP-vehicle or GFP::PKM2 (n=3 per group). **(L)** Apoptosis competence measured by the Luminescence Caspase 3/7-Glo assay (n=2 per group, one-way ANOVA, DF: 7, F-value 6.496, p-value 0.04). Significance: unpaired t-test, *<0.05, **<0.01.

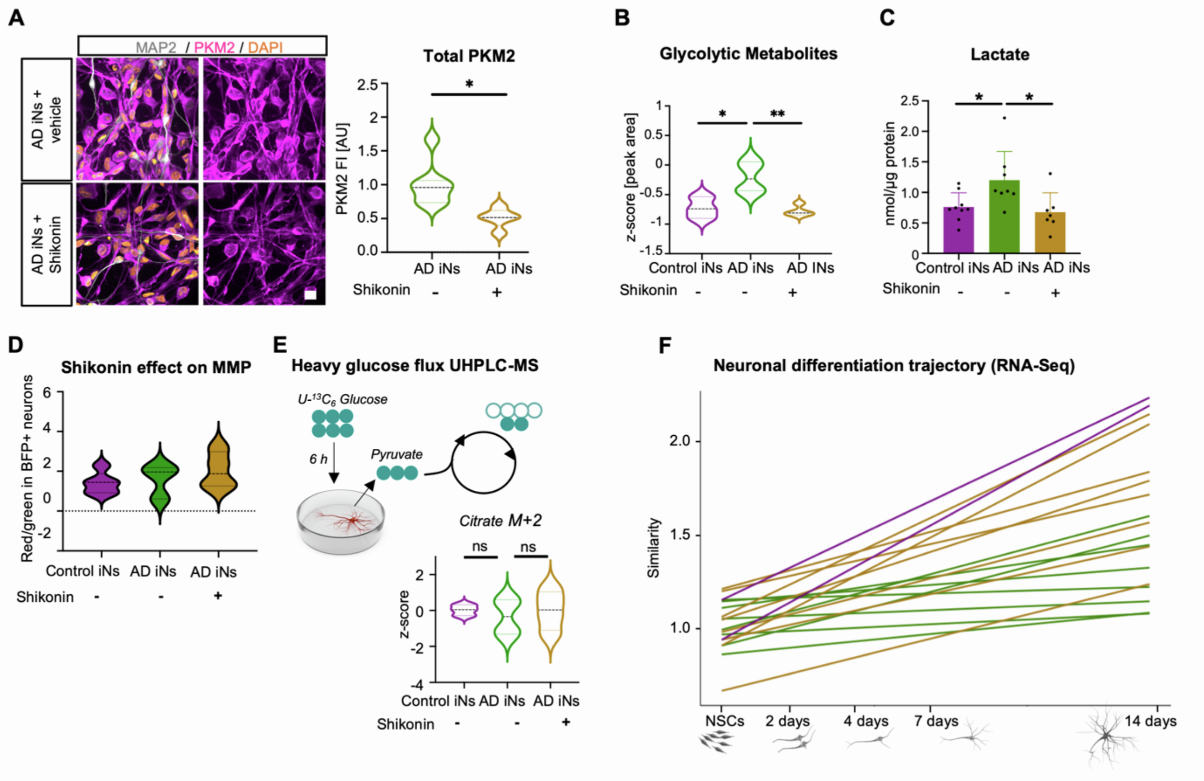

Figure S7. Shikonin restores mature neuronal phenotype

Related to Figure 7. **(A)** Immunostaining and quantification of total PKM2 FI in MAP2-ROIs in AD iNs with and without shikonin (vehicle n=6, shikonin n=5, unpaired t-test). Scale bar = 10 µm. **(B)** Average of all detected glycolytic metabolites measured by UHPLC-MS-based metabolomics (DF:10, F-value 10.12, p-value 0.0064). **(C)** Colorimetric assay measuring lactate levels in supernatant of control (n=9), AD (n=8) and shikonin-treated AD (n=7) iNs normalized to total protein (DF:23, F-value 5.087, p-value 0.01). **(D)** Mitochondrial membrane potential of iNs treated with and without shikonin measured by JC-1 in PSA-NCAM$^+$ neurons (n=3 per group, DF:11, F-value 0.78, p-value 0.48). **(E)** Tracing of isotope-labeled glucose after six hours of incubation with $^{13}C_6$-Glucose. Fraction of labeled glucose detected in citrate (n=10 per group, DF:10, F-value 0.18, p-value 0.8). **(F)** Correlation of trajectory genes in control (n=3), AD (n=8) and AD + shikonin (n=8) iNs to neuronal differentiation from NSCs to neurons (Schafer et al. 2019) for each patient. **(B-E)** Significance: one-way ANOVA *<0.05, **<0.01.
