# Supplementary material for: Warburg-like metabolic transformation underlies neuronal degeneration in sporadic Alzheimer’s disease
Source: Cell Metab. 2022 Sep 6;34(9):1248–1263.e6. doi: 10.1016/j.cmet.2022.07.014 (PMC9458870; doi:10.1016/j.cmet.2022.07.014)

Figure 1D

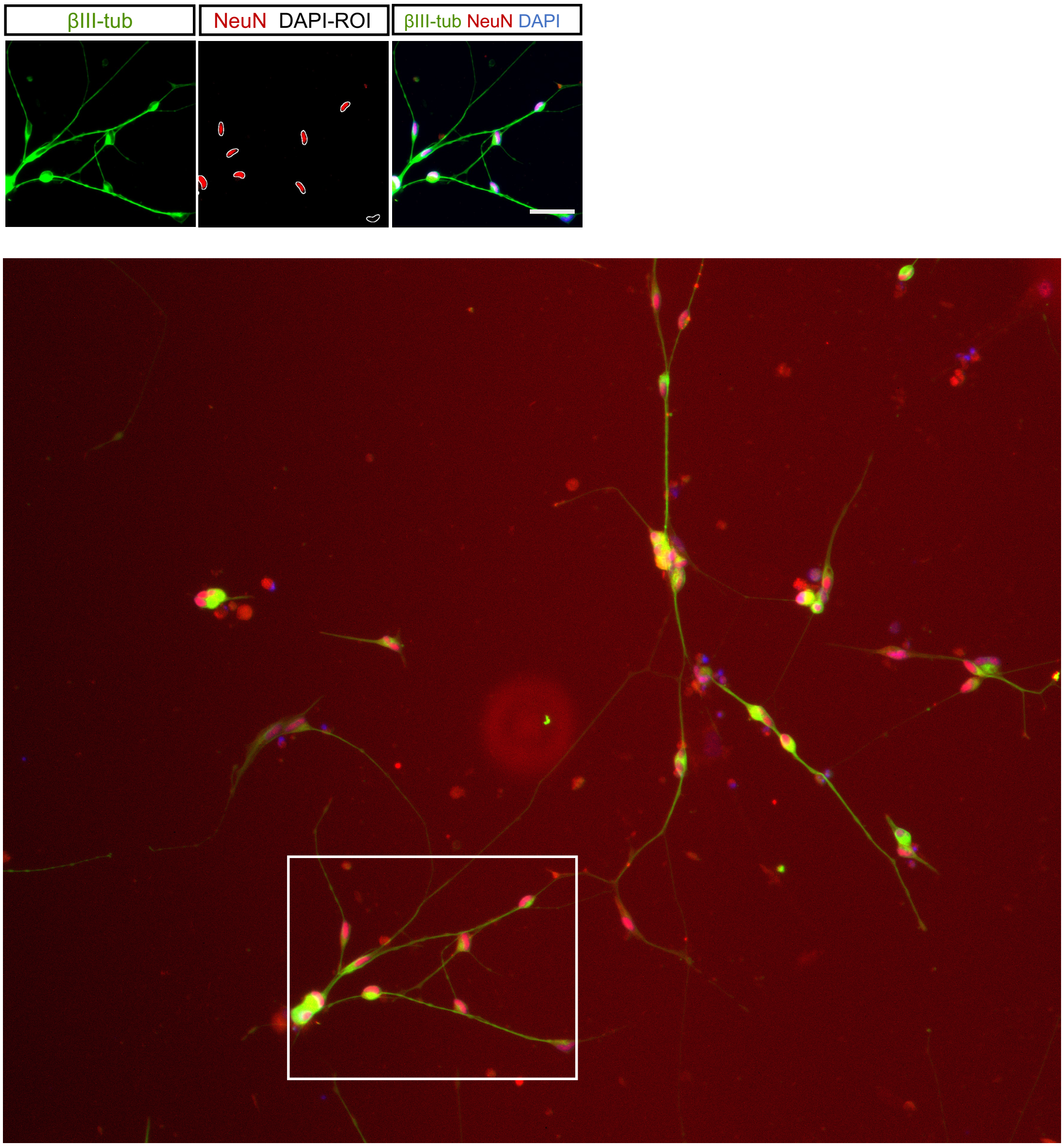

Figure 6A

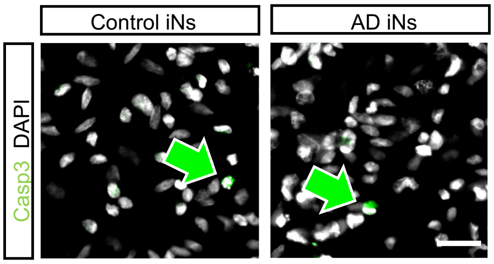

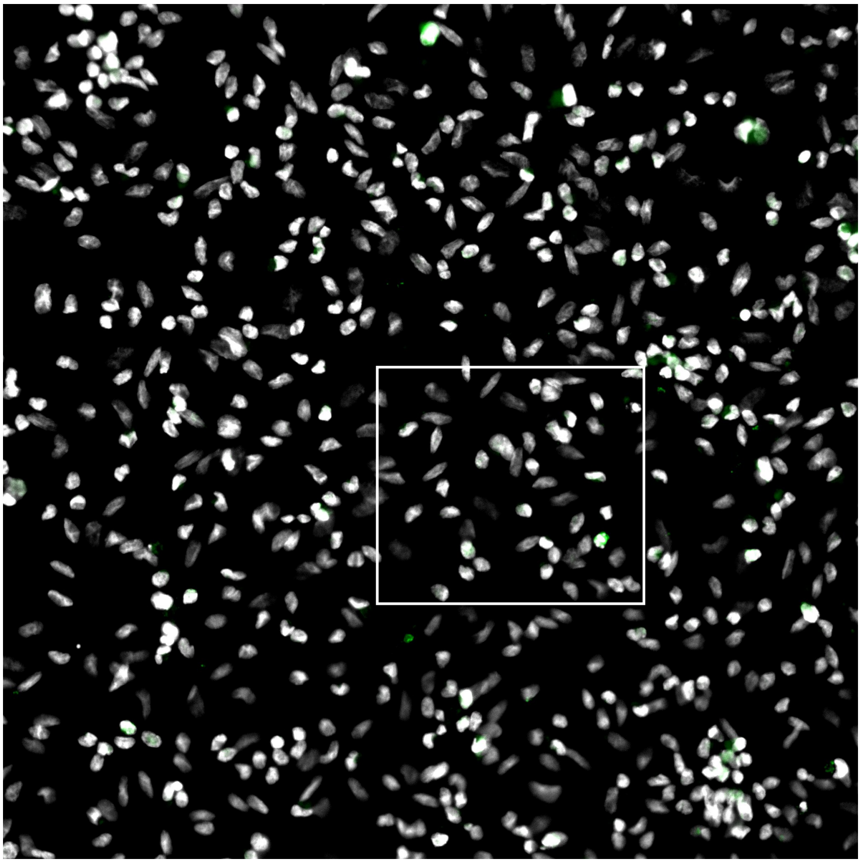

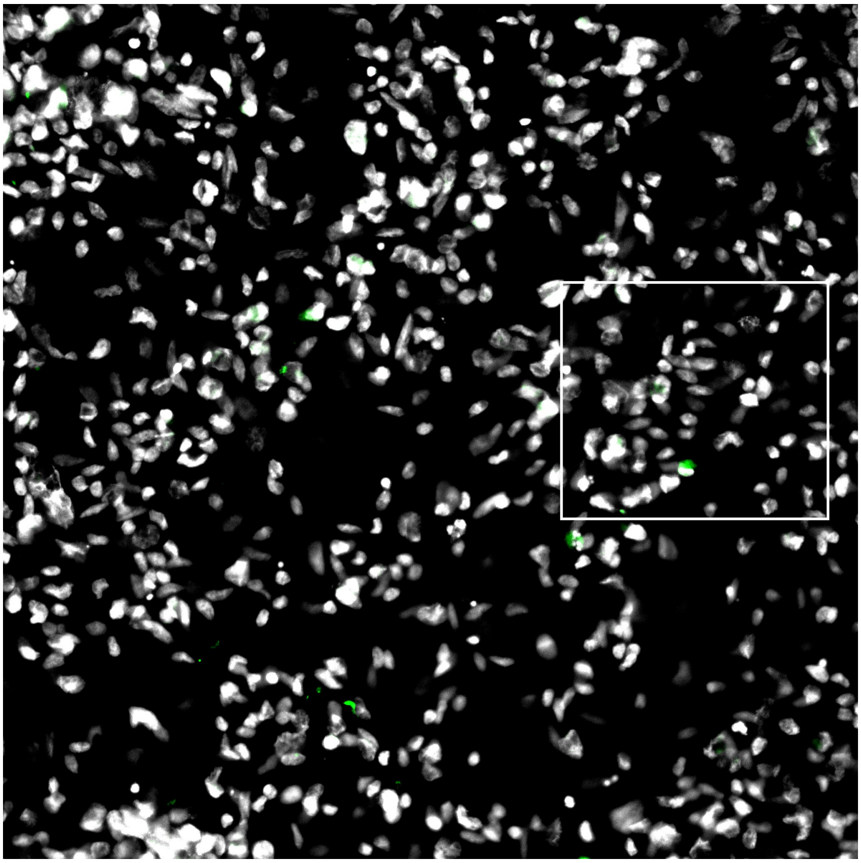

Figure 6E

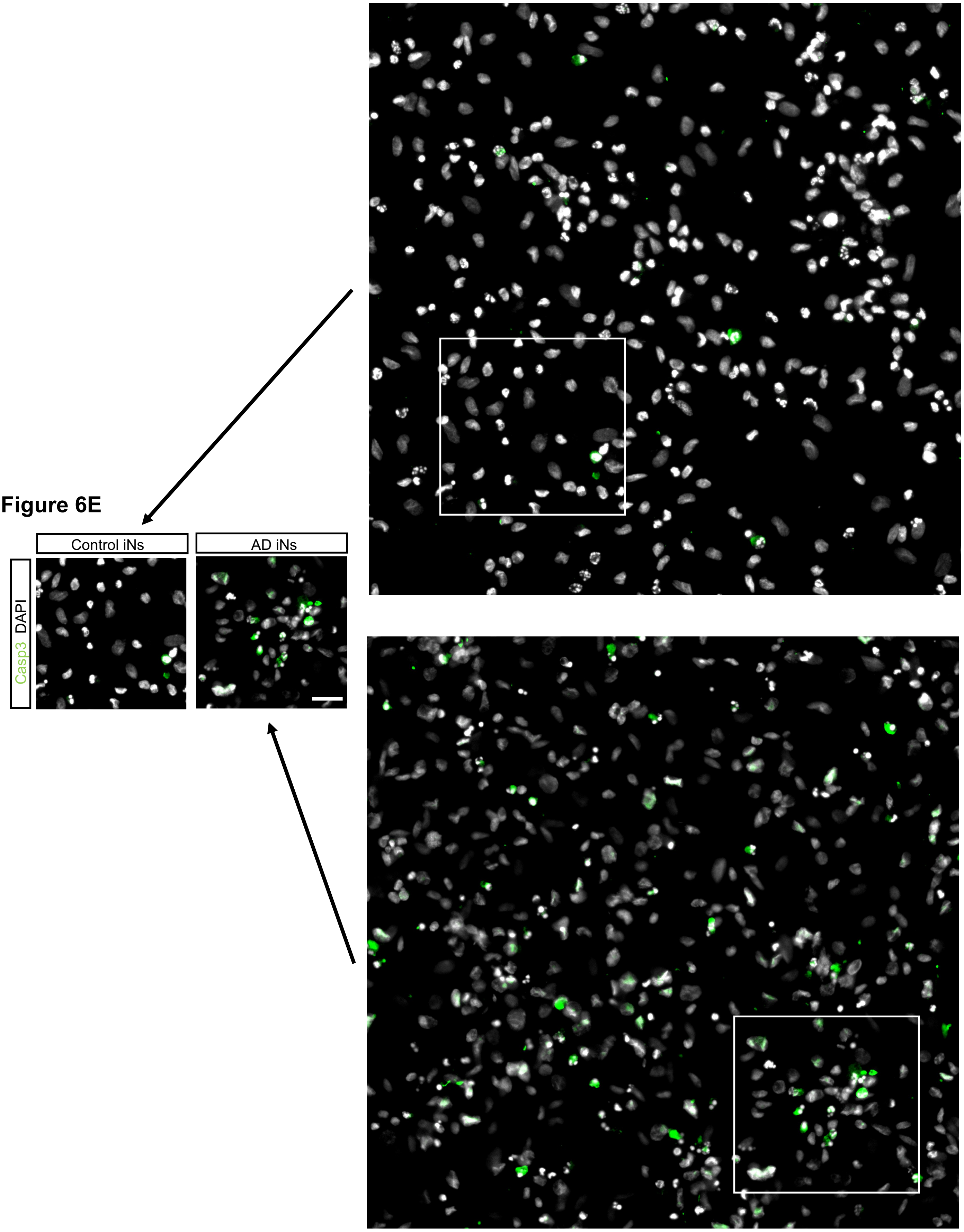

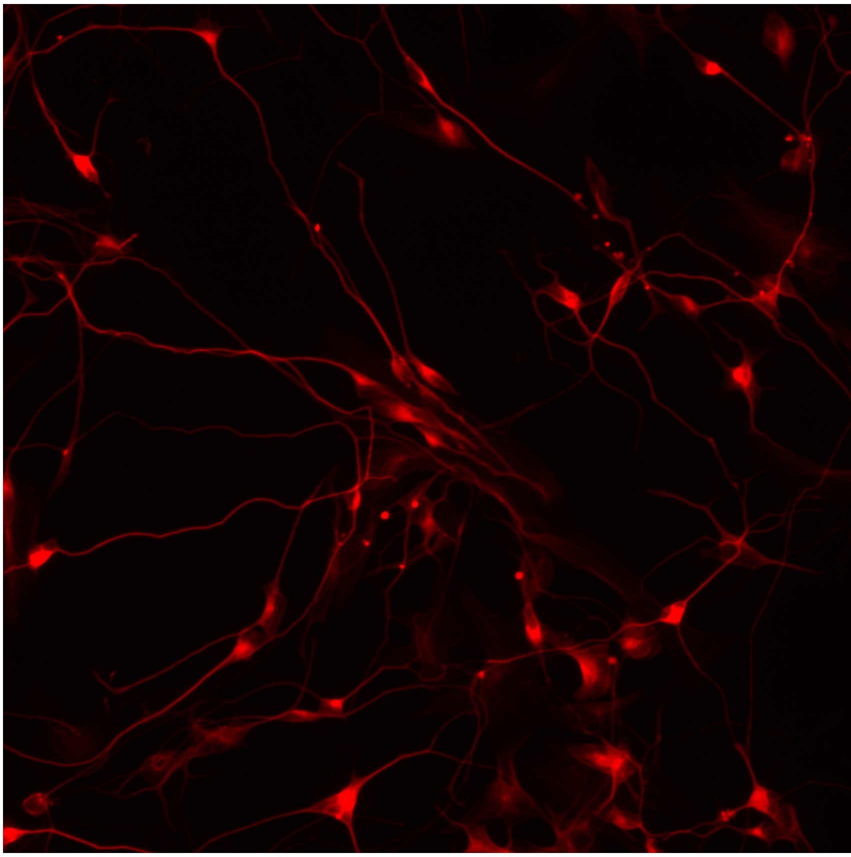

Figure 6K

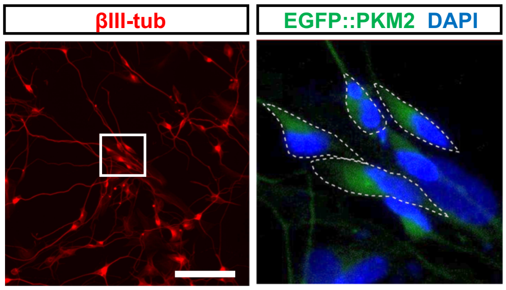

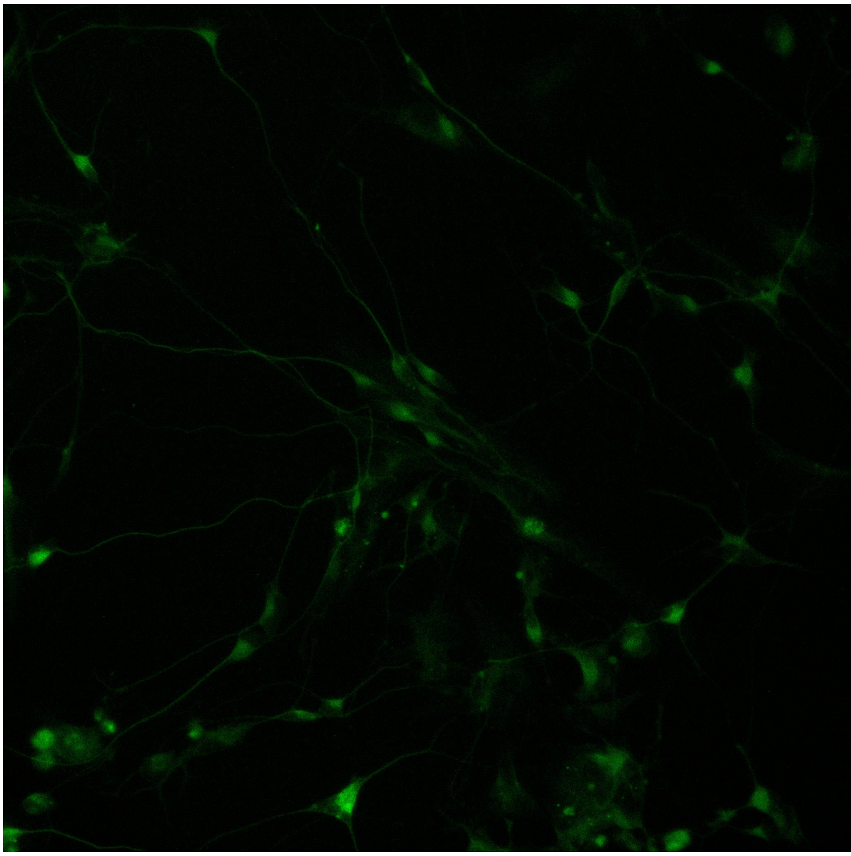

Figure 6M

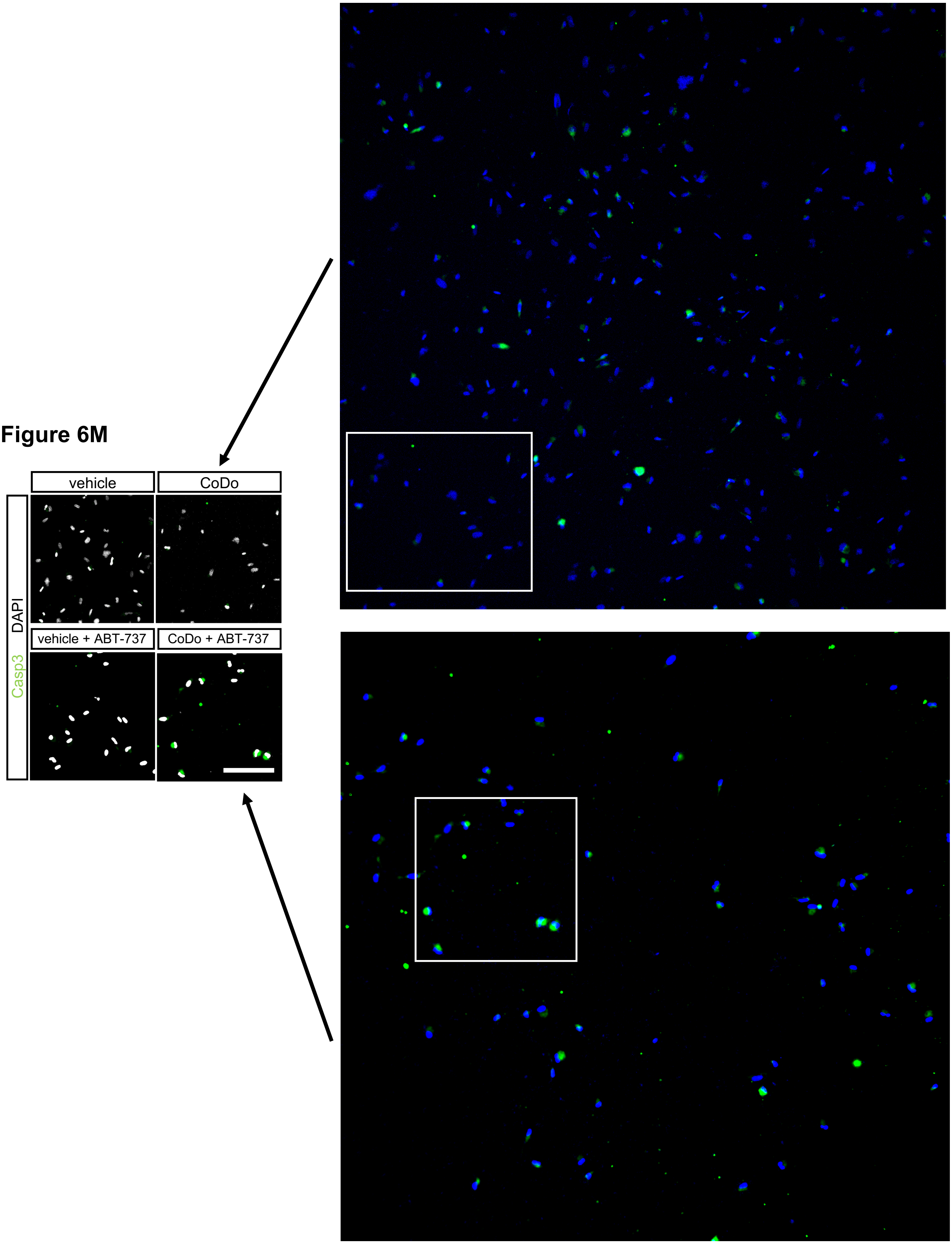

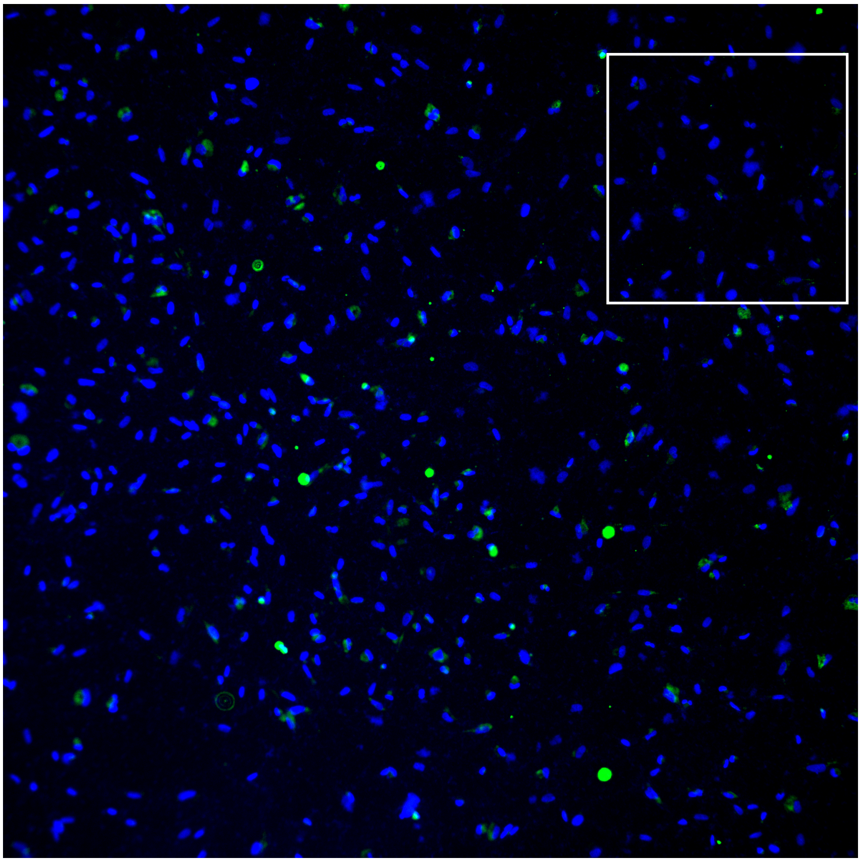

Figure 6M

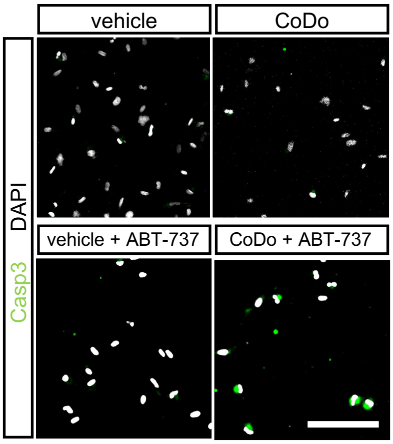

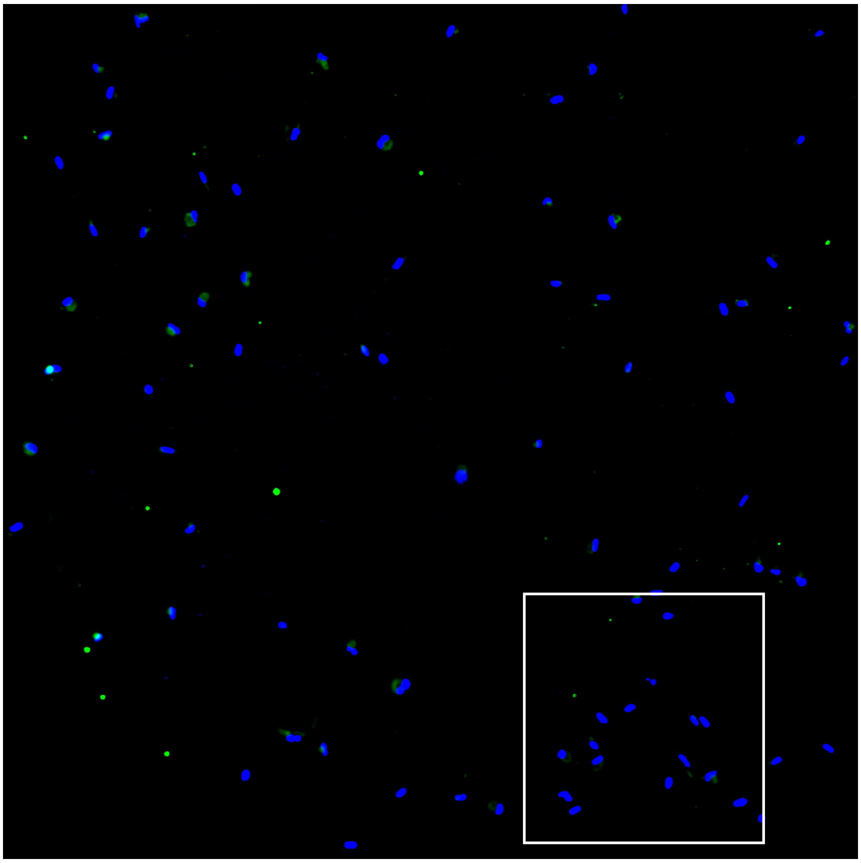

Figure 7L

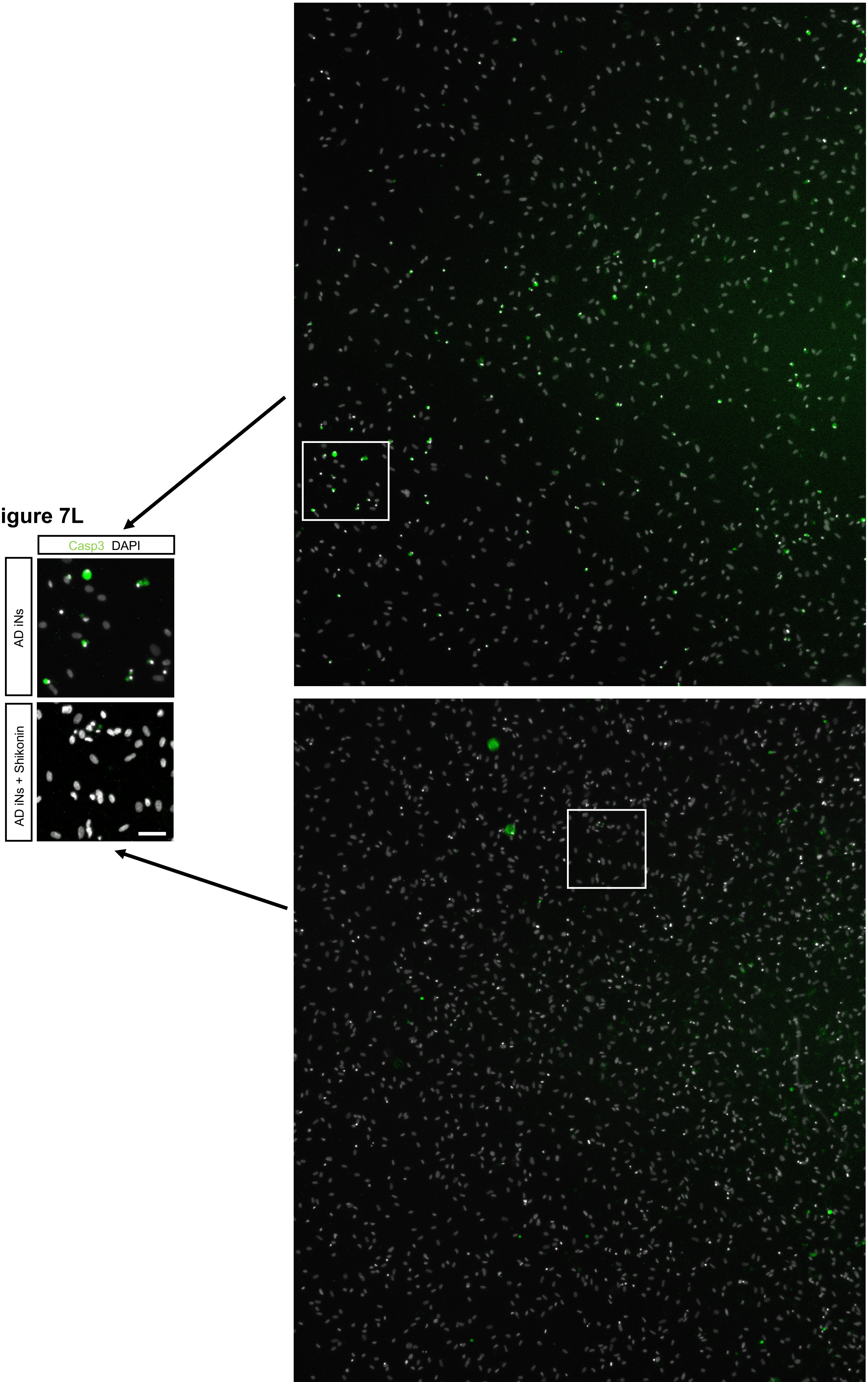

Figure S1B

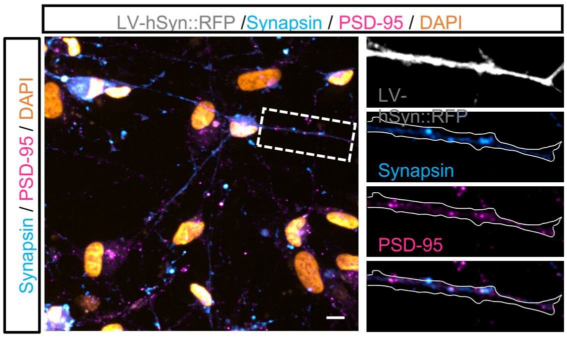
LV-hSyn::RFP /Synapsin / PSD-95 / DAPI
Synapsin / PSD-95 / DAPI
LV-hSyn::RFP
Synapsin
PSD-95

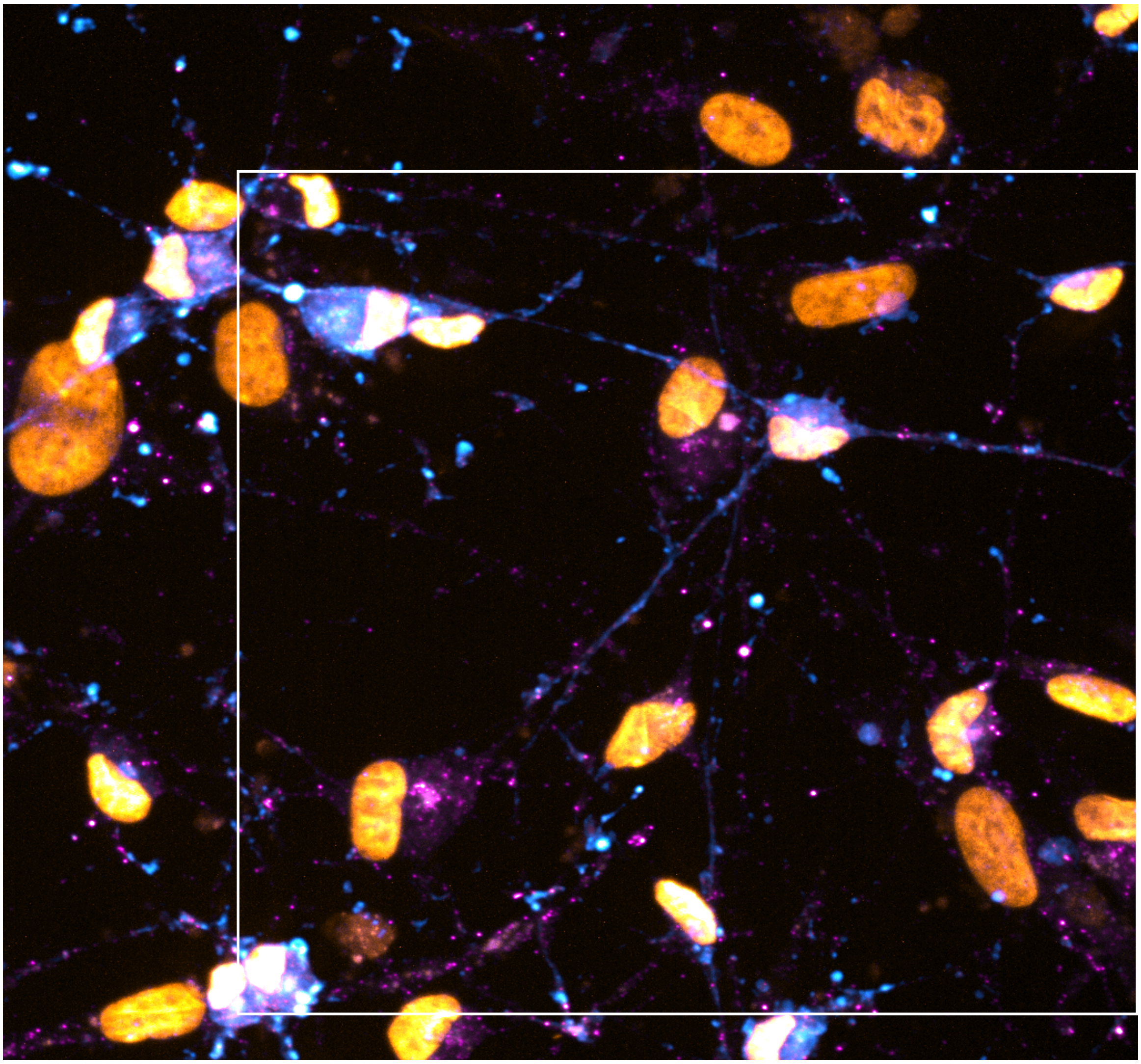

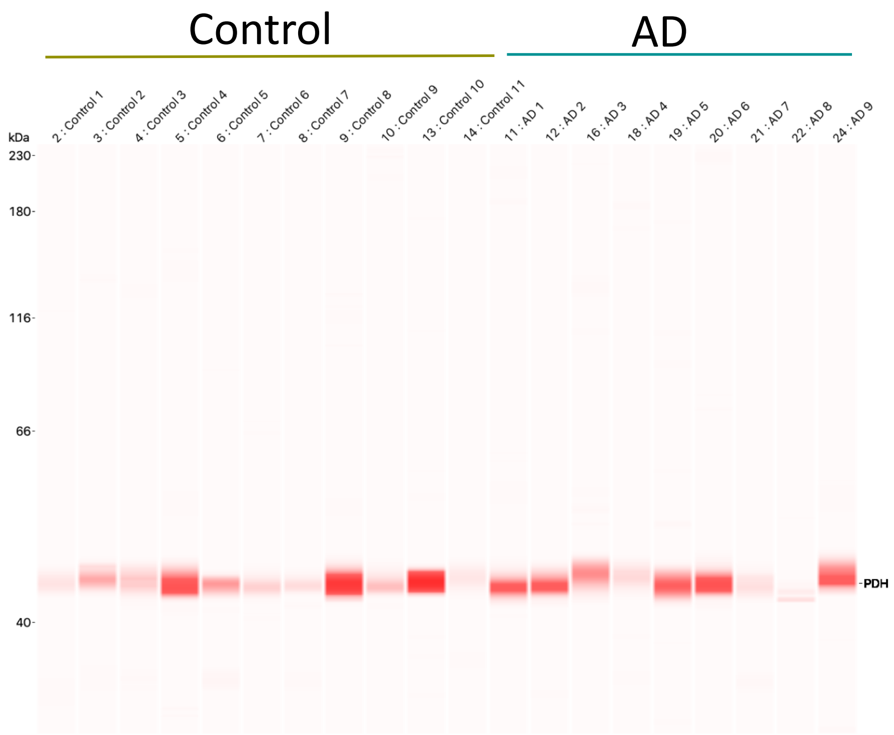

Figure S4D. PDH protein level

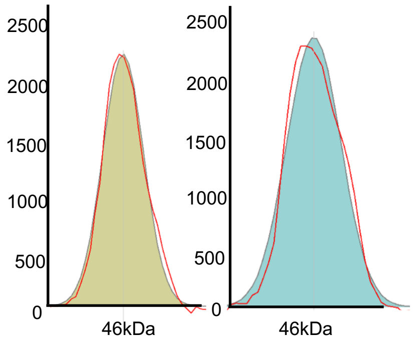

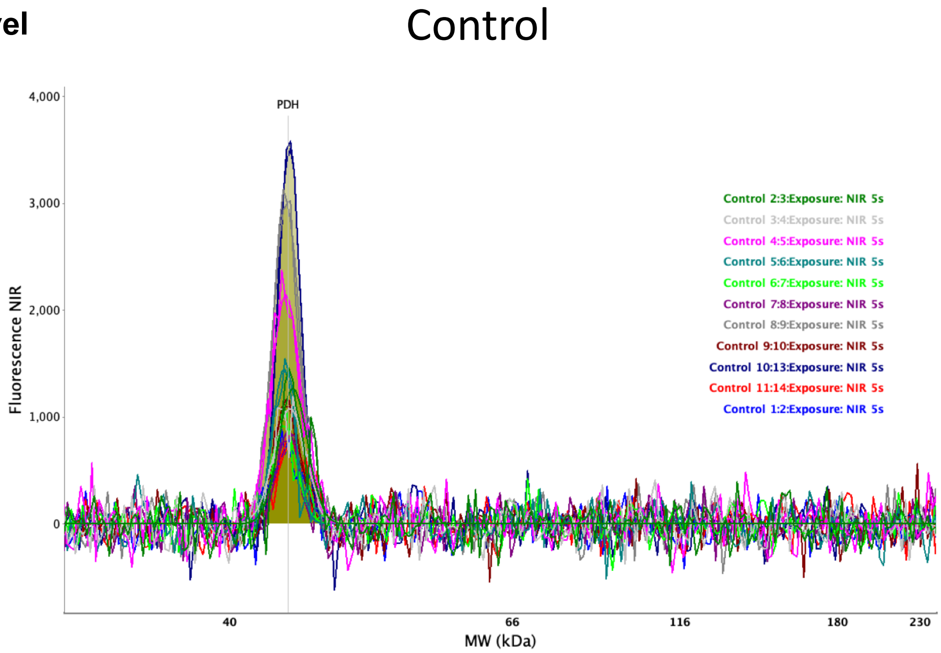

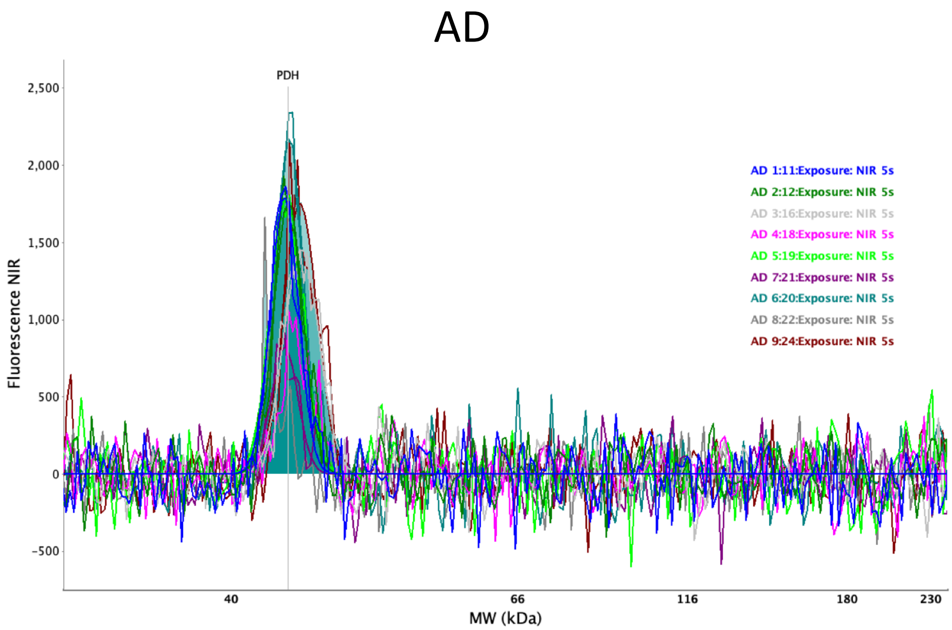

Supplement: Data S1. Unprocessed data underlying the display items in the manuscript — Provides uncropped images of immunofluorescence analyses related to Figures 1D, 6A, 6E, 6K, 6M, 7L, and S1B and uncropped blots of Simple western Blot analysis related to Figure S4D. Additionally, unprocessed data related to all main and supplemental Figures are provided. [file mmc2.zip › Data S1 - Source Data/Figures DataS1.pdf]
